# Residential exposure to transportation noise and risk of incident atrial fibrillation: a pooled study of 11 prospective Nordic cohorts

**DOI:** 10.1016/j.lanepe.2024.101091

**Published:** 2024-10-01

**Authors:** Jesse D. Thacher, Nina Roswall, Mikael Ögren, Andrei Pyko, Agneta Åkesson, Anna Oudin, Annika Rosengren, Aslak H. Poulsen, Charlotta Eriksson, David Segersson, Debora Rizzuto, Emilie Helte, Eva M. Andersson, Gunn Marit Aasvang, Gunnar Engström, Hrafnhildur Gudjonsdottir, Jenny Selander, Jesper H. Christensen, Jørgen Brandt, Karin Leander, Kim Overvad, Kristoffer Mattisson, Kristina Eneroth, Lara Stucki, Lars Barregard, Leo Stockfelt, Maria Albin, Mette K. Simonsen, Ole Raaschou-Nielsen, Pekka Jousilahti, Pekka Tiittanen, Petter L.S. Ljungman, Steen S. Jensen, Susanna Gustafsson, Tarja Yli-Tuomi, Thomas Cole-Hunter, Timo Lanki, Youn-Hee Lim, Zorana J. Andersen, Göran Pershagen, Mette Sørensen

**Affiliations:** a Division of Occupational and Environmental Medicine, Lund University, Lund, Sweden; b Danish Cancer Institute, Strandboulevarden 49, 2100, Copenhagen Ø, Denmark; c Department of Occupational and Environmental Medicine, Sahlgrenska University Hospital, Gothenburg, Sweden; d Occupational and Environmental Medicine, School of Public Health and Community Medicine, Institute of Medicine, University of Gothenburg, Gothenburg, Sweden; e Institute of Environmental Medicine, Karolinska Institutet, Stockholm, Sweden; f Center for Occupational and Environmental Medicine, Region Stockholm, Stockholm, Sweden; g Division of Sustainable Health, Umeå University, Sweden; h Molecular and Clinical Medicine, Sahlgrenska Academy, University of Gothenburg, Gothenburg, Sweden; i Region Västra Götaland, Sahlgrenska University Hospital, Gothenburg, Sweden; j Swedish Meteorological and Hydrological Institute, Norrköping, Sweden; k Aging Research Centre, Department of Neurobiology, Care Sciences and Society, Karolinska Institutet and Stockholm University, Stockholm, Sweden; l Stockholm Gerontology Research Centre, Stockholm, Sweden; m Department of Air Quality and Noise, Norwegian Institute of Public Health, Oslo, Norway; n Department of Clinical Science, Lund University, Malmö, Sweden; o Centre for Epidemiology and Community Medicine, Stockholm, Sweden; p Department of Global Public Health, Karolinska Institutet, Stockholm, Sweden; q Department of Environmental Science, Aarhus University, Roskilde, Denmark; r Department of Public Health, Aarhus University, Aarhus, Denmark; s Environment and Health Administration, Stockholm, Sweden; t Department of Neurology and Parker Institute, Bispebjerg and Frederiksberg Hospital, Denmark; u Department of Public Health and Welfare, Finnish Institute for Health and Welfare, Helsinki, Finland; v Department of Health Security, Finnish Institute for Health and Welfare, Kuopio, Finland; w Department of Cardiology, Danderyd Hospital, Stockholm, Sweden; x Environmental Department of the City of Malmö, Malmö, Sweden; y Section of Environmental Health, Department of Public Health, University of Copenhagen, Copenhagen, Denmark; z School of Medicine, University of Eastern Finland, Kuopio, Finland; aa Department of Environmental and Biological Sciences, University of Eastern Finland, Kuopio, Finland; ab Department of Natural Science and Environment, Roskilde University, Denmark

**Keywords:** Atrial fibrillation, Arrhythmia, Air pollution, Cardiac, Pooled cohort, Road traffic noise, Railway noise, Aircraft noise

## Abstract

**Background:**

Transportation noise has been linked with cardiometabolic outcomes, yet whether it is a risk factor for atrial fibrillation (AF) remains inconclusive. We aimed to assess whether transportation noise was associated with AF in a large, pooled Nordic cohort.

**Methods:**

We pooled data from 11 Nordic cohorts, totaling 161,115 participants. Based on address history from five years before baseline until end of follow-up, road, railway, and aircraft noise was estimated at a residential level. Incident AF was ascertained via linkage to nationwide patient registries. Cox proportional hazards models were utilized to estimate associations between running 5-year time-weighted mean transportation noise (L_den_) and AF after adjusting for sociodemographics, lifestyle, and air pollution.

**Findings:**

We identified 18,939 incident AF cases over a median follow-up of 19.6 years. Road traffic noise was associated with AF, with a hazard ratio (HR) and 95% confidence interval (CI) of 1.02 (1.00–1.04) per 10-dB of 5-year mean time-weighted exposure, which changed to 1.03 (1.01–1.06) when implementing a 53-dB cut-off. In effect modification analyses, the association for road traffic noise and AF appeared strongest in women and overweight and obese participants. Compared to exposures ≤40 dB, aircraft noise of 40.1–50 and > 50 dB were associated with HRs of 1.04 (0.93–1.16) and 1.12 (0.98–1.27), respectively. Railway noise was not associated with AF. We found a HR of 1.19 (1.02–1.40) among people exposed to noise from road (≥45 dB), railway (>40 dB), and aircraft (>40 dB) combined.

**Interpretation:**

Road traffic noise, and possibly aircraft noise, may be associated with elevated risk of AF.

**Funding:**

10.13039/501100004785NordForsk.

**Research in context**

**Evidence before this study**

We searched PubMed (until June 15, 2024) for studies on traffic noise and atrial fibrillation incidence. We used the search terms: “atrial fibrillation” OR “arrhythmia” OR “cardiac arrhythmia” AND “noise exposure” OR “traffic noise” OR “community noise” OR “traffic noise exposure” OR “road traffic noise” OR “road noise” OR “rail traffic noise” OR “rail noise” OR “rail traffic noise” OR “railway noise” OR “air traffic noise” OR “aircraft noise” with no language or time restrictions.

To date there are five studies on road traffic noise, two on railway noise, and three on aircraft noise and atrial fibrillation. Although some of these studies have found associations, findings are inconsistent across studies. The existing studies have either been limited by sample size, accuracy of noise modelling, or covariate availability. Despite growing awareness of the deleterious health effects of noise, there is a lack of literature pertaining to atrial fibrillation.

**Added value of this study**

This is the first multi-national prospective study of traffic noise and atrial fibrillation incidence. Our large study with harmonized cohort data on socioeconomic and lifestyle covariates and atrial fibrillation, together with complete address history for all participants linked with noise exposure using comparable high-quality noise models across cohorts, have allowed us to investigate the association with road traffic, railway, and aircraft noise without the limitations encountered in previous similar studies.

We found that long-term residential exposure to road traffic and aircraft noise seemed associated with higher risk of atrial fibrillation, whereas no association was observed for railway noise. For road traffic and aircraft noise, we observed indications of exposure-response relationships. The associations remained following adjustment for various socioeconomic and lifestyle factors, as well as for air pollution. Exposure to multiple transportation noise sources seemed particularly harmful. We also found that individuals with high BMI and unhealthy lifestyle habits seemed more susceptible to the harmful effect of road traffic noise on atrial fibrillation development.

**Implications of all the available evidence**

The EU estimation of the health burden from traffic noise in 2020 did not include atrial fibrillation due to lack of evidence. Although more studies on noise and atrial fibrillation are needed, our results suggest that the disease burden associated with road traffic noise and potentially aircraft noise may be underestimated when not including atrial fibrillation.

## Introduction

Atrial fibrillation (AF), the most prevalent sustained cardiac arrhythmia, impacting around 4% of persons over the age of 50 years.^1^ AF is characterized by an irregular and often rapid heart rhythm, and increases the risk of stroke, thromboembolism, and other cardiovascular complications.^2^ Age, smoking, being overweight or obese, sleep apnea, and having a sedentary lifestyle are all risk factors for AF. Furthermore, some studies have suggested that environmental exposures, such as traffic noise and air pollution, might be associated with AF eitiology.^3^, ^4^, ^5^

Transportation noise is a major environmental health problem globally. It is estimated that at least 20% of the European population is exposed to transportation noise at levels higher than the Environmental Noise Directive recommended noise limit of 55 dB L_den_ (noise over the whole day).^6^ This is likely underestimated as only urban areas with over 100,000 residents and major roads with over three million vehicles per year, major railway tracks, and major airports are included.^6^ As both traffic noise exposure and AF are frequent, identifying whether transportation noise is linked with risk of AF is important.

Epidemiological studies have linked transportation noise with an elevated risk of cardiometabolic outcomes including hypertension,^7^ ischemic heart disease,^8^ stroke,^9^ and type 2 diabetes,^10^^,^^11^ but the association with AF remains inconclusive. To date, five cohort studies investigated the association between road traffic noise exposure and AF incidence.^3^, ^4^, ^5^^,^^12^^,^^13^ Three Danish studies demonstrated that long-term road traffic noise was associated with incident AF,^3^, ^4^, ^5^ while two studies from Sweden and England found no association.^12^^,^^13^ For railway noise, a Danish study comprising the entire adult population observed a positive association between railway noise and incident AF,^3^ while in a cohort of 50,373 Danes no association was observed.^5^ Three studies have investigated aircraft noise exposure and AF: a Danish national study found an association with AF in highly exposed individuals,^3^ a Greek study suggested aircraft noise at night to be associated with cardiac arrhythmia,^14^ and in a German cohort, aircraft noise annoyance was associated with higher odds of AF.^15^

Chronic exposure to traffic noise is thought to negatively impact health through various pathways such as via stress, annoyance, and sleep disturbance.^16^ More specifically, repeated activation of the sympathetic-adrenal-medulla (SAM) and hypothalamus-pituitary-adrenal (HPA) axes elicit the secretion of stress hormones, which precipitates various physiological effects, including increased heart rate, changes in electrophysiology, elevated blood pressure and viscosity, as well as the activation of blood coagulation.^10^ Furthermore, disturbed sleep is a purported risk factor for AF, and sleep deprivation and fragmentation are associated with endothelial dysfunction, impaired immune system, and increased levels of pro-inflammatory molecules.^10^ This suggests that sleep disturbance may be an important mechanistic pathway from noise exposure to AF. Collectively these pathways could contribute to the etiology of cardiovascular diseases, such as AF.

In the present study, we aimed to examine the association between residential levels of road, railway, and aircraft noise and incident AF in a pooled study of 11 cohorts from Nordic countries.

## Methods

### Study participants

The NordSOUND project (“Nordic Studies on Occupational and Traffic Noise in Relation to Disease”) was initiated to explore the health impacts of noise (www.nordsound.dk). The study population included 11 cohort studies from Nordic countries: eight Swedish, two Danish, and one Finnish. All centers harmonized exposure, outcome, and covariate data according to a predefined codebook and data was checked for compliance with this codebook following upload to the central database.

A detailed overview of each participating cohort has been described earlier and is presented in Supplemental Table S1.^17^^,^^18^ Briefly, two cohorts were based in Denmark: the Danish Nurse Cohort (DNC; nationwide) and the Diet, Cancer, and Health cohort (DCH; from Aarhus and Copenhagen). Four Swedish cohorts situated in Stockholm County, the Stockholm Screening Across the Lifespan Twin Study (SALT), the Stockholm 60 years old study (Sixty), the Swedish National Study of Aging and Care in Kungsholmen (SNAC-K), and the Stockholm Diabetes Prevention Programme (SDPP). These four studies comprised the “Cardiovascular Effects of Air pollution and Noise Study” (CEANS) and employed the same methodology to assess environmental exposures and standardized covariate data.^19^ Four additional Swedish studies were also involved: the Malmö Diet and Cancer study (MDCS; Malmö), the Swedish Mammography cohort (SMC; Uppsala), the Multinational Monitoring of Trends and Determinants in Cardiovascular Diseases cohort (GOT-MONICA; Gothenburg), and the Swedish Primary Prevention Cohort (PPS; Gothenburg). Finally, we included a Finnish cohort originating from the cities of Helsinki, Vantaa, and Turku (FINRISK).^20^

Each cohort had residential address histories and were register annually for most Swedish cohorts, except for the SMC cohort which had exact date of address change. Similarly, precise date of address change was also recorded for the Danish and Finnish cohorts. Where necessary, start of follow-up for the study participants was postponed guaranteeing participants had a minimum of five years of transportation noise exposure information preceding the start of follow-up (referred to as the NordSOUND baseline).

The initial cohort studies were approved by all respective institutional medical ethics committees and were completed in accordance with the Declaration of Helsinki.

### Definition of atrial fibrillation

The ascertainment of AF before and during follow-up was accomplished through linkage to Swedish, Danish, and Finnish nationwide patient registers, recognized for their high validity and comprehensiveness.^21^^,^^22^ The Danish patient register contains records from both inpatients (since 1987) and outpatients (since 1995). In Sweden, the patient registry also contains records from inpatients (since 1987) and outpatients (since 2001). However, only the Swedish MDCS cohort obtained data from both in- and outpatients, whereas the remaining Swedish cohorts relied on inpatient records only. No outpatient records were available in Finland. Individuals with AF prior to the NordSOUND baseline were excluded. We defined incident AF as the first principal or secondary discharge diagnosis based on the International Classification of Diseases (ICD) 8 codes 427.92, 427.93, or 427.94, ICD9 codes 427D, 427.31, or 427.32, or ICD10 code I48.

### Exposure assessment

The NordSOUND study's methodology for estimating road, railway, and aircraft noise exposure has been previously detailed.^9^ In short, transportation noise at residential addresses was calculated at the most exposed façade as the equivalent continuous A-weighted sound pressure levels (L_Aeq_), and expressed as L_den_. Five- and ten-dB penalties were added to evening (defined as 19–22 in Denmark and Finland and 18–22 in Sweden) and night (defined as 22–07 in Denmark and Finland and 22–06 in Sweden), respectively, accounting for higher sensitivity to noise in these intervals. Annual road and railway noise were estimated for GOT-MONICA and PPS, every five years for the DNC, DCH, Sixty, SDPP, SNAC-K, SMC, and SALT cohorts, and every ten years for the MDCS cohort, and for FINRISK the 2011 noise levels were utilized. We employed linear interpolation in all cohorts except MDCS, to estimate noise exposure levels for intermediate years, and to estimate yearly mean (logarithmic average) noise exposure estimates from five years preceding enrollment to end of follow-up. For MDCS, annual exposure was determined by selecting the year closest in proximity to the modeled data, or the year when significant infrastructure modifications occurred.

In all cohorts, the Nordic prediction method was employed to model road traffic noise (Supplemental Table S2).^23^ Input variables for the model comprised geocoded addresses, screening from terrain or buildings, yearly mean daily traffic, ground absorption, allotment of light and heavy vehicles, speed of traffic, as well as road type for all principal roads. For smaller roads (roads with <1000 vehicles per day), traffic information was present for all cohorts besides FINRISK and the Stockholm cohorts, and data for noise barriers was present in the Danish, Gothenburg, and Finnish cohorts.

The Nordic prediction method, or an updated version of this method, Nord2000, was utilized to estimate railway noise (Supplemental Table S3).^24^ Noise from railways was estimated for addresses within ≤1000 m to a railway line (all cohorts except SMC, in which no railway noise was estimated), metro (Stockholm, Copenhagen, and Helsinki), or tramlines (Stockholm, Gothenburg, and Helsinki). Model input variables comprised geocoded address, the mean frequency of trains per period (day, evening, night), speed and type of train, ground absorption, as well as screening by buildings or terrain. Residences were considered unexposed to railway noise if situated >1000 m from a rail, tramline, or metro.

Road and railway noise exposure was modeled as time-weighted means (logarithmic mean) for the previous one and five years and accounted for address history in the respective intervals. If a missing period was greater than 20% of the interval length, we imputed missings by taking the average from the whole interval.

Road and railway noise levels below L_den_ 40 dB were set to 40 dB since this is the assumed level of background noise from other sources as well as inaccuracy of noise estimates below this level.^9^

For the Stockholm cohorts, aircraft noise was estimated in one dB categories from noise maps constructed by Swedavia utilizing the Integrated Noise Model 7.0. Aircraft noise for 2011 was modelled for FINRISK based on ECAC Doc 29, 3rd edition in five dB categories greater than 50 dB. In the DNC and DCH cohorts, aircraft noise was estimated in five dB categories using noise maps created by regional authorities for separate airfields and airports and employing the Danish Airport Noise Simulation Model as well as the Integrated Noise Model. Few participants were estimated to be exposed to aircraft noise in the MDCS, GOT-MONICA, PPS, and the SMC cohorts due to flight routes, and therefore this was not assessed. In the pooled analyses, aircraft noise was categorized (L_den_ ≤40, >40–50, >50 dB) since some cohorts lacked a more detailed exposure assessment.

### Covariates

Covariate selection was completed *a priori* considering availability, current literature, biological plausibility, and ability to harmonize across cohorts (Supplemental Figure S1).

At enrollment, each participant completed a questionnaire which queried information on lifestyle, physical activity during leisure time (low, medium, high), alcohol intake (never, seldom, daily, or weekly; missing for PPS), smoking (current, former, or never), smoking intensity (grams of tobacco per day; missing for PPS), and body mass index (BMI; kilograms/meters,^2^ which was completed by qualified staff during recruitment for all cohorts except DNC, SALT, and SMC in which self-reports were used). Marital status (married or cohabiting, living independently) and level of education (high, medium, or low) were gathered using questionnaires or national registers, while income (in quartiles based on country-specific income distribution) at the area level (homogenous areas with 1000–2000 inhabitants) was obtained from registers.

During the study period, annual average concentrations of particulate matter with an aerodynamic diameter of ≤2.5 μm (PM_2.5_) as well as nitrogen dioxide (NO_2_) were assessed for all addresses from high-resolution dispersion models (see Supplemental Table S4 for details).

### Statistical analysis

Cox proportional hazards regression models, using age as the underlying timescale, were utilized to calculate AF hazard ratios (HRs) per 10 dB L_den_ higher levels of road and railway noise. Each participant was followed from the NordSOUND baseline and was right censored at the age of an AF diagnosis, loss to follow-up, emigration, over 20% missing in exposure-periods, death, or end of follow-up (December 31, 2011–December 31, 2017, depending on the cohort), whichever came first. Exposure to noise was modelled as time-weighted 1- and 5-year running means, taking exposure at all addresses in the period into account (including moving), and entered as time-varying variables into the Cox model.

For railway noise models, an indicator variable denoting any exposure >40 dB versus none was also included with the continuous variable. Aircraft noise was investigated in categories: ≤40 (reference), >40–50, and >50 dB. The GOT-MONICA, PPS, MDCS, and SMC cohorts did not have information on aircraft noise and were omitted when calculating the association with aircraft noise.

The assumption of linearity of the associations for continuous variables was assessed by cubic splines with four degrees of freedom. We observed deviation from linearity for BMI, thus a piecewise linear function with a knot at 22 kg/m^2^ was included. We tested the proportional hazards assumption of the Cox Models using a Pearson correlation test between the scaled Schoenfeld residuals and the rank order of event time. We detected deviation from the assumption for calendar year, smoking status, and sex, and therefore, all analyses were completed with these variables as strata. To allow for different baseline hazards across cohorts, all models were stratified by cohort.

Correlations between the exposure variables were examined by Spearman's rank correlation coefficients.

The association between transportation noise and AF was investigated in four predefined models: Model 1, adjusted for age (by design), sex, and calendar year (in 5-year categories); Model 2, further adjustment for education level, marital status, and area-level income, and mutual transportation noise adjustment (road traffic noise, linear; railway noise, linear and category for exposed yes/no; aircraft noise in categories, 40, >40–50, >50 dB); Model 3, additional adjustment for physical activity and smoking status; and Model 4, with further adjustment for PM_2.5_. We *a priori* considered model 3 as our primary model. Models 1–3 were conducted on complete data for all covariates (except aircraft noise), while model 4, was confined to persons with complete PM_2.5_ exposure information. In addition, HRs were calculated considering exposure to zero (reference), one, two, or three transportation noise sources of ≥45 dB, >40 dB, and >40 dB for road, railway, and aircraft noise, respectively. The cut point of 40 dB for railway and aircraft noise was selected as people exposed to ≤40 dB were assumed unexposed in the current study. For road traffic noise, only very few were exposed to ≤40 dB, especially in the urban cohorts, and therefore we used a cut point of 45 dB to define people with low exposure.

We also assessed the association between road traffic noise and AF including a cut-off at 53 dB (i.e., setting those with road traffic noise levels below 53 dB–53 dB). This value was selected as the WHO, in their recently published noise guidelines, concluded that road traffic noise greater than 53 dB was linked with negative health outcomes.^25^

The influence of additional adjustment for alcohol consumption (daily, weekly, seldom, never), smoking intensity (g/day), BMI, as well as for NO_2_ in cohorts with data was investigated.

Cohort-specific risks between road, rail, and aircraft noise exposure and AF in our primary model (Model 3) as well as leave-one-out analyses, omitting each cohort individually were completed.

We evaluated possible effect modification of the association between road traffic noise and incident AF by including a multiplicative interaction term between possible effect modifiers and the 5-year mean noise exposure and calculated p-values for interaction using the Wald test. This included sex, age at diagnosis, BMI (<25 kg/m^2^ [normal], 25–29.99 kg/m^2^ [overweight], ≥30 kg/m^2^ [obese]), educational level, smoking status, physical activity, PM_2.5_, exposed to railway noise (≤and >53 dB), population density, having a previous diagnosis of ischemic heart disease (ICD9: 410–414 and ICD10: I20–I25, entered as time-varying), and diagnosis of AF using out-patient data or not.

Analyses were completed using SAS (version 9.4, SAS Institute, Cary, NC) and R (version 3.2.2).

### Role of the funding sources

The funders of the study had no role in study design, data collection, data analysis, data interpretation, writing of the report, or in the decision to submit the paper for publication.

## Results

Out of the 179,966 participants comprising the original cohorts, we omitted 1589 with AF at NordSOUND baseline or earlier, 2231 with missing exposure data in the five years before baseline, and 15,031 with missing covariates, resulting in 161,115 individuals in the present study. Among these, 18,939 incident AF cases were diagnosed over a median follow-up for the population of 19.6 years.

Table 1 presents the baseline characteristics of the study population stratified by exposure to road traffic noise. Overall, highly exposed individuals (≥60 dB) were more often single, and more likely to smoke and drink alcohol daily compared to those less exposed (<50 dB). Baseline characteristics stratified by cohort are shown in Supplemental Table S5.

**Table 1:** Characteristics of the study population at the NordSOUND baseline by 5-year mean baseline road traffic noise.

	Total	Road traffic noise exposure		
		<50 dB	50–60 dB	≥60 dB
**N**^a^	161,115	80,516	35,759	44,840
**Atrial fibrillation cases, N**	18,939	8208	4555	6176
**Age at study baseline, y**	55.9 (45.0–72.0)	55.2 (44.3–71.0)	56.3 (45.7–72.4)	56.7 (46.0–72.8)
**Sex, %**				
Men	34.3	33.8	33.7	35.6
Women	65.7	66.2	66.3	64.4
**Educational level, %**				
Low	32.6	31.5	33.5	33.9
Medium	48.2	49.4	46.9	47.1
High	19.2	19.1	19.6	19.1
**Marital status, %**				
Married/cohabiting	72.9	78.3	70.7	65.2
**Area–level income, %**				
1st quartile	25.9	27.5	25.9	23.0
2nd quartile	21.0	20.4	22.1	21.2
3rd quartile	21.9	21.1	23.0	22.5
4th quartile	31.2	31.0	29.0	33.3
**Smoking status, %**				
Current	31.1	28.9	31.7	34.7
Former	30.3	30.9	29.7	29.7
Never	38.6	40.2	38.6	35.6
**Smoking intensity, g/day**^b^^**,**^^c^	14.4 (3.0–30.0)	13.8 (3.0–30.0)	14.5 (3.0–30.0)	15.0 (3.0–30.0)
**Physical activity, %**				
Low	39.3	38.5	38.2	41.6
Medium	31.6	32.9	30.9	29.8
High	29.1	28.6	30.9	28.6
**BMI (kg/m**^**2**^**)**	24.9 (19.9–32.7)	24.8 (20.0–32.5)	24.9 (19.9–32.7)	25.0 (19.9–33.0)
**Alcohol intake, %**^b^				
Daily	13.9	12.9	14.0	15.5
Weekly	51.0	52.2	49.8	49.9
Seldom	25.5	25.5	26.0	25.1
Never	9.6	9.4	10.1	9.5
**Population density, %**				
Low	14.5	19.0	10.6	9.6
Medium	27.8	30.2	29.4	22.4
High	57.4	50.8	60.0	68.0

Median and 5–95 percentiles, unless otherwise stated.

a Number of participants with full information on exposure variables, outcome, and all covariates in adjustment Model 3.

b Not available for all cohorts.

c Among current smokers.

The mean 5-year road traffic noise exposure at baseline varied from 46.0 to 62.2 dB across the cohorts (Table 2). Railway and aircraft noise levels were somewhat lower than road traffic noise, with baseline median noise levels ranging from 46.3 to 53.1 dB for railway noise. Only 1.8% of the individuals were exposed to aircraft noise over 50 dB. Mean baseline PM_2.5_ concentrations were highest in the Danish cohorts (19.8–20.9 μg/m^3^), while the Swedish and Finnish cohorts had lower concentrations (7.6–13.7 μg/m^3^). We observed low to moderate correlations between 5-year road traffic noise and other exposures, with the highest correlations between road traffic noise and NO_2_ (R_Spearman_ of 0.49) (Table 3). We found a very high correlation between road traffic Lden and Lnight (R_Spearman_ of 0.998).

**Table 2:** Baseline exposure to transportation noise (L_den_) and air pollution across the included cohorts.

	DCH	DNC	SDPP	Sixty	SNAC–K	SALT	MDCS	PPS	GOT–MONICA	SMC	FINRISK	Total
**Road traffic noise, 5–y, dB**	56.9 (0.7)	53.9 (0.8)	46.0 (0.6)	51.3 (0.8)	62.2 (0.7)	51.9 (0.9)	54.9 (0.8)	58.2 (0.8)	55.9 (0.8)	54.6 (0.8)	54.4 (0.8)	55.0 (0.8)
**Railway noise, % exposed**^a^	25.9	19.1	14.4	31.6	51.9	34.5	28.1	15.2	19.6	–	34.2	22.7
**Railway noise, 5–y, dB**^b^	52.9 (0.7)	53.1 (0.7)	51.9 (0.7)	50.2 (0.7)	49.7 (0.6)	50.3 (0.7)	49.2 (0.8)	47.0 (0.6)	46.3 (0.5)	–	49.4 (0.7)	51.3 (0.8)
**Aircraft noise, 5–y, %**												
≤40 dB	98.6	98.8	74.7	83.6	15.2	83.3	–	–	–	–	95.6	95.6
40.1–50 dB	0.7	0.4	9.6	13.4	65.3	13.5	–	–	–	–	0.6	2.6
>50 dB	0.7	0.8	15.7	3.0	19.5	3.2	–	–	–	–	3.8	1.8
**PM**_**2.5**_**, 5–y, μg/m**^**3**^	19.8 (1.9)	20.9 (3.4)	7.6 (0.6)	8.1 (0.9)	8.5 (0.8)	7.7 (0.9)	11.0 (0.8)	–	9.3 (1.4)	13.7 (0.8)	7.6 (0.8)	15.4 (5.5)
Missing, %	0.04	9.0	0	0	0	0	0.02	100^c^	0	0.01	0.06	4.7
**NO**_**2**_**, 5–y, μg/m**^**3**^	29.3 (8.1)	13.3 (8.1)	8.8 (2.8)	14.1 (6.7)	21.1 (4.8)	14.2 (6.5)	24.4 (6.4)	31.4 (6.2)	29.2 (7.1)	8.5 (5.9)	16.3 (4.5)	21.1 (10.8)
Missing, %	0.04	9.0	0	0	0	0	0.02	2.5	0	0.01	0.06	1.6

Values given as mean and standard deviation (SD) unless otherwise stated.

a Exposed in the 5-year period preceding baseline.

b Among exposed to railway noise.

c PPS only had information on PM_2.5_ from baseline until follow–up.

**Table 3:** Spearman correlations between traffic noise and air pollution exposures.

	1-y road traffic noise	5-y road traffic noise	1-y railway noise	5-y railway noise	1-y aircraft noise^a^	5-y aircraft noise^a^	1-y PM_2.5_^b^	5-y PM_2.5_^b^	1-y NO_2_^b^	5-y NO_2_^b^
1-y road noise	–									
5-y road noise	0.96	–								
1-y railway noise	0.12	0.12	–							
5-y railway noise	0.12	0.13	0.94	–						
1-y aircraft noise^a^	−0.05	−0.05	−0.03	−0.03	–					
5-y aircraft noise^a^	−0.06	−0.06	−0.02	−0.03	0.95	–				
1-y PM_2.5_^b^	0.27	0.26	0.01	0.02	−0.21	−0.21	–			
5-y PM_2.5_^b^	0.29	0.27	0.01	0.01	−0.22	−0.22	0.96	–		
1-y NO_2_^b^	0.48	0.48	0.19	0.19	−0.15	−0.16	0.47	0.42	–	
5-y NO_2_^b^	0.48	0.49	0.19	0.20	−0.15	−0.15	0.40	0.39	0.98	–

All exposures are calculated as 1- and 5-year time-weighted means before baseline taking all addresses in the periods into account.

a Only in cohorts with aircraft noise (thus excluding MDCS, PPS, GOT-MONICA, and SMC), N = 109,112.

b Only among subjects with PM_2.5_ and NO_2_ data (N = 153,717 and 158,817, respectively).

**Fig. 1:**
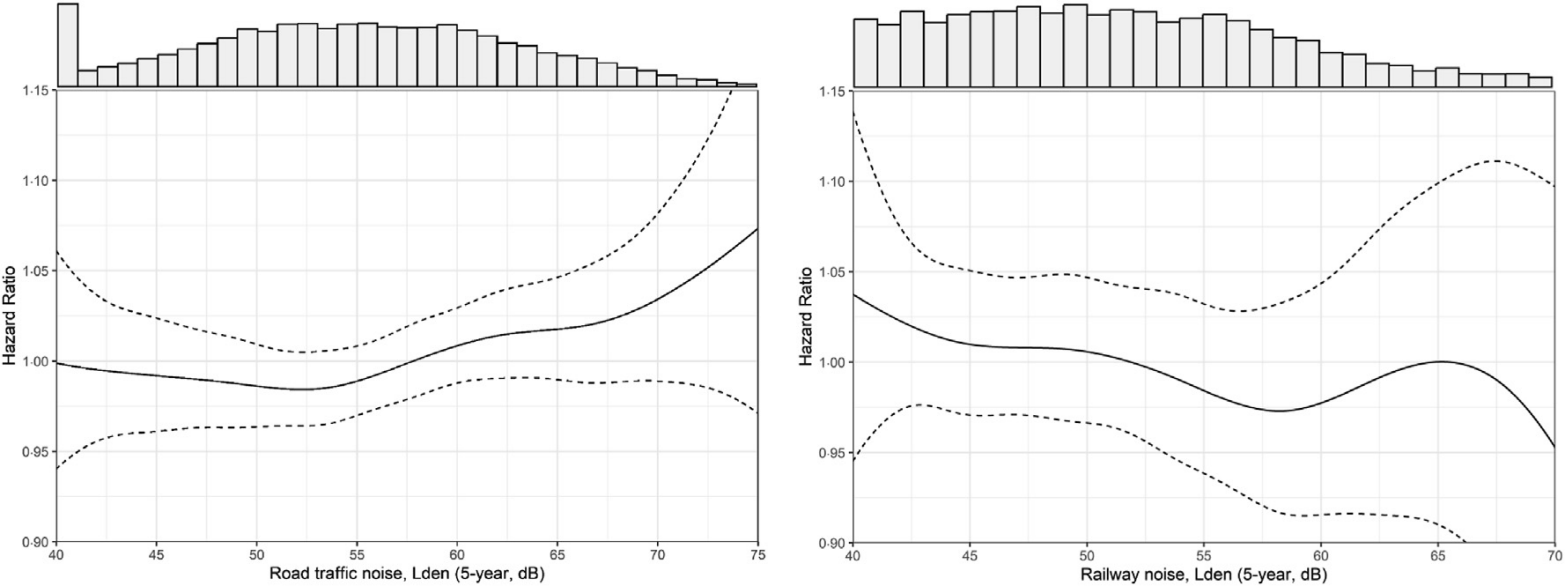
**Exposure-response relationship between 5-year mean exposure to road traffic and railway noise and risk for atrial fibrillation in models adjusted for age, cohort, sex, calendar-year, educational level, marital status, area-income, other noise sources, smoking status, and physical activity. The distribution of road traffic noise is shown for the whole population and for railway noise for the 22.7% of the population exposed**.

Road traffic noise was associated with a 2% higher hazard ratio of incident AF (95% confidence interval (CI): 0–4%) per 10 dB higher 5-year exposure (Table 4). Adjustment for individual lifestyle factors as well as particulate air pollution resulted in only minor reductions in HRs (Table 4, Supplement Table S6). The HRs for 1-year mean road traffic noise were similar to the 5-year mean in adjustment model 3, with a HR of 1.02 (1.00–1.04) (Supplemental Table S7). Effect estimates began to monotonically increase at around 53 dB and followed a roughly linear exposure-response relationship above this level (Fig. 1). Using the >53 dB as a cut-off, we found a slightly stronger association for AF, with a HR of 1.03 (95% CI: 1.01–1.06) per 10 dB (Table 4). Overall, no clear heterogeneity was observed in risk estimates for AF between cohorts as illustrated by overlapping CIs (Supplemental Table S8). The omission of any single cohort resulted in minor differences in HRs in leave-one-cohort-out analyses (Supplemental Table S9).

**Table 4:** Association between 5-year mean exposure to transportation noise and risk of atrial fibrillation.

	N cases	Model 1^a^ HR (95% CI)	Model 2^b^ HR (95% CI)	Model 3^c^ HR (95% CI)	Model 4^d^ HR (95% CI)
**Road traffic noise, L**_**den**_					
5-year exposure, per 10 dB	18,939	1.03 (1.01–1.05)	1.02 (1.00–1.04)	1.02 (1.00–1.04)	1.02 (1.00–1.04)
5-year exposure, per 10 dB with 53 dB cut–off	12,424 ≥53 dB	1.05 (1.02–1.08)	1.04 (1.01–1.06)	1.03 (1.01–1.06)	1.03 (1.00–1.06)
**Railway noise, L**_**den**_					
5-year exposure, per 10 dB	18,939	0.98 (0.94–1.02)	0.97 (0.93–1.01)	0.97 (0.93–1.01)	0.98 (0.94–1.02)
**Aircraft noise, L**_**den**_**, 5-year**^e^					
≤40 dB	11,100	Reference	Reference	Reference	Reference
40.1–50 dB	513	1.03 (0.93–1.16)	1.04 (0.93–1.16)	1.04 (0.93–1.16)	1.04 (0.93–1.17)
>50 dB	313	1.10 (0.97–1.25)	1.11 (0.98–1.26)	1.12 (0.98–1.27)	1.12 (0.99–1.27)

HR: Hazard Ratio; 95% CI: 95% Confidence Interval.

a Model 1: adjusted for age, cohort (strata), sex and calendar year.

b Model 2: Model 1 plus adjustment for educational level, marital status, area–income, and other noise sources (road, railway and aircraft noise), for the four cohorts without aircraft noise information, all cohort members were assigned to the ≤40–dB group).

c Model 3: Model 2 plus adjustment for smoking status and physical activity.

d Model 4: Model 3 plus adjustment for time–weighted PM_2.5_ exposure (1– or 5-year). PM_2.5_ exposure history available for 17,999 cases.

e Only among cohorts with aircraft noise exposure, including a total of 11,765 cases.

All main results of the present paper are based on complete case analyses. In Supplemental Table S10 we report that HRs were unchanged in analyses including cohort participants excluded from the main analyses due to missing information on education (N = 4767), smoking (N = 1999) and/or physical activity (N = 4487) to our study population. No association between railway noise and AF incidence was observed, irrespective of the time-window or level of adjustment (Table 4). Also, no exposure-response association was indicated for railway noise when results were presented with a smoothing spline (Fig. 1).

For aircraft noise, there was no clear association, although there was some indication for the >50 dB group (HR 1.12 (95% CI: 0.98–1.27) (Table 4). Cohort-specific analyses are presented in Supplemental Table S8.

Dichotomizing exposure from each source of traffic noise into exposed/unexposed (defined as ≥45 dB from road, >40 dB from railway and >40 dB for aircraft noise) we found that HRs were higher as number of exposures increased (Supplemental Table S11). Compared to those without any elevated traffic noise exposure, HRs (95% CI) for exposures to one, two, and three noise sources were 1.04 (0.98–1.10), 1.06 (1.00–1.13), and 1.19 (1.02–1.40), respectively.

**Fig. 2:**
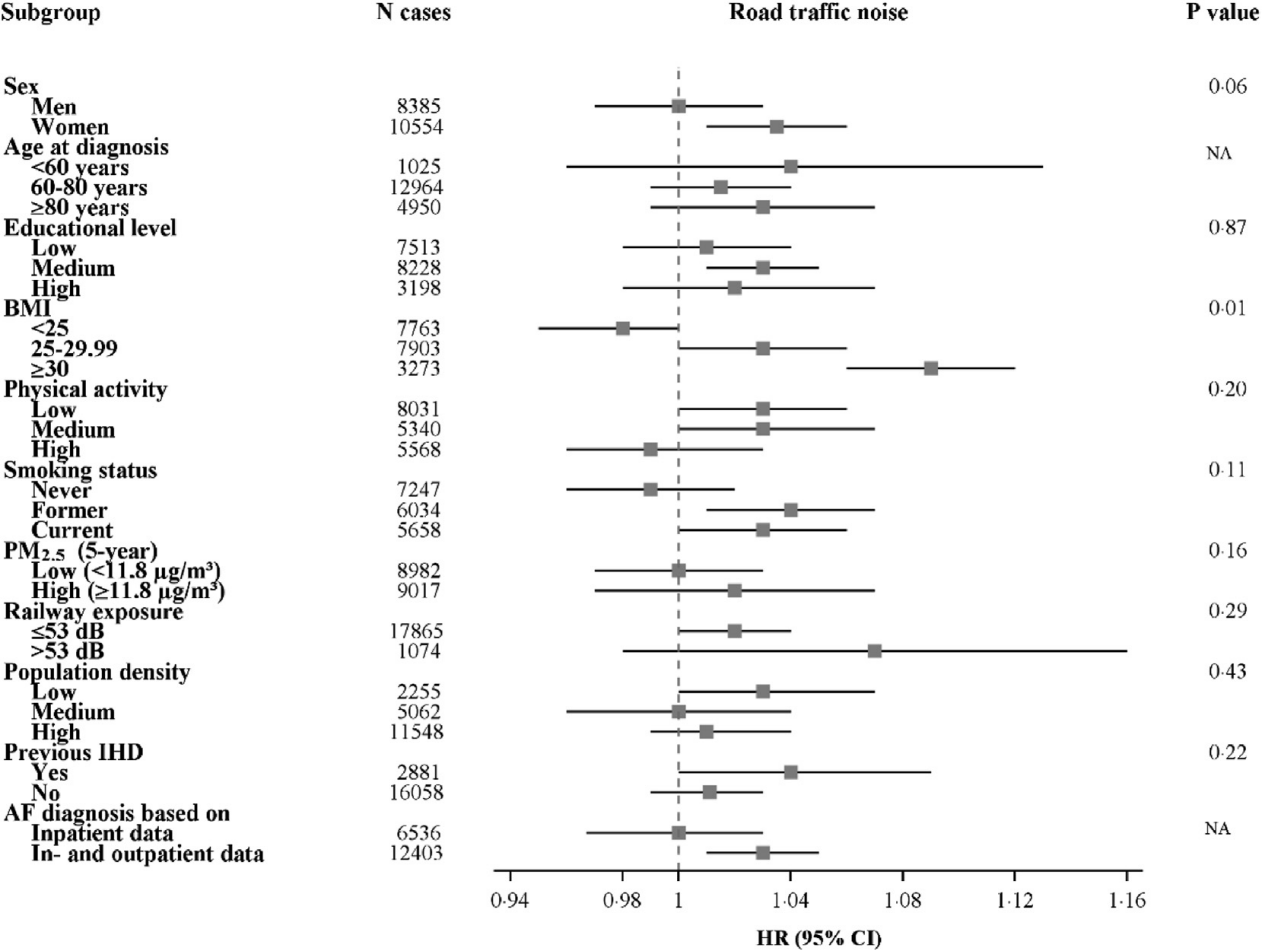
**Interactions between 5-year mean exposure to road traffic noise and demographic factors, lifestyle factors, categories of air pollution and railway noise, population density, previous ischemic heart disease (IHD), and type(s) of register information used to identify cases, in relation to risk of atrial fibrillation. Models adjusted for age, cohort, sex, calendar-year, educational level, marital status, area-income, railway and aircraft noise, smoking status, and physical activity**.

No apparent modification of the association between road traffic noise and AF by age, education, and population density was observed (Fig. 2, Supplemental Table S12). Nonetheless, we found somewhat stronger associations among females (P_interaction_ = 0.06), people with high BMI (P_interaction_ ≤0.01), and possibly former or current smokers (P_interaction_ = 0.11), people with low or medium physical activity (P_interaction_ = 0.20), people with high exposure to PM_2.5_ (P_interaction_ = 0.16), people with a previous diagnosis of ischemic heart disease (P_interaction_ ≤0.22), and cohorts identifying AF based on both in- and outpatient data (compared to only inpatient data). Similar patterns were seen when effect modification was assessed using a cut-off of 53 dB (Supplemental Table S13).

For all three sources of traffic noise, further adjustment for particulate air pollution, BMI, smoking intensity, and alcohol consumption only resulted in very small changes of the observed HRs. For road traffic noise, the HR (95% CI) changed from 1.02 (1.00–1.04) to 1.01 (0.99–1.04) following adjustment for NO_2_, whereas no change was observed in analyses using the >53 dB cut-off, with a HR of 1.03 both before and after adjustment for NO_2_ (Supplemental Table S6).

## Discussion

In this pooled study with data from 11 cohorts from Sweden, Denmark, and Finland, long-term exposure to residential road traffic and potentially aircraft noise, seemed associated with a higher risk of incident AF. For both exposures, we observed indications of exposure-response relationships. The associations remained controlling for various sociodemographic and lifestyle covariates and particulate air pollution. Residential exposure to railway noise was not associated with incident AF. HRs were higher with exposure to a higher number of transportation noise sources. We found somewhat stronger associations between road traffic noise and AF in women and individuals with a BMI ≥30 kg/m^2^.

This is one of the largest studies on transportation noise and incident AF including data on individual sociodemographic and lifestyle covariates. We found that road traffic noise was associated with a slightly higher risk of AF irrespective of time-window of exposure, with an indication of a threshold around the WHO guideline limit (53 dB), below which there was no apparent association with road traffic noise, suggesting that only noise levels above this cut-off may be harmful in relation to AF development. When we accounted for this cut-off in the analyses, the risk associated with road traffic noise was slightly stronger from a HR of 1.02 to 1.03, suggesting that examining the shape of the exposure-response function to investigate whether a lower threshold exists is important in noise studies. However, another potential explanation is lower precision of the estimation of noise at low noise levels compared to higher noise levels.

Three of the 11 cohorts in the present study have reported earlier on road traffic noise and incident AF, with two of them, the DCH and the DNC, reporting positive associations with road traffic noise with HRs of 1.06 and 1.03, respectively,^4^^,^^5^ whereas the PPS cohort found no association.^12^ Importantly, in the present study we have for DCH and the DNC included longer follow-up time compared to the previous studies, resulting in 4230 and 679 additional cases, respectively.^4^^,^^5^ Of the remaining existing literature, a Danish nationwide cohort study found a weak association for road traffic noise, although only for noise at the least exposed façade (an indicator for bedroom exposure as individuals often choose to sleep in a room with a silent façade), with a HR of 1.013 (95% CI: 1.007–1.019).^3^ In contrast, a cohort of 200,000 adults from London found no association between road traffic noise at night and AF incidence.^13^ However, in the London study, exposure was derived from postal codes, not exact residential addresses; therefore, the study was more prone to exposure misclassification than the Nordic studies, which relied on exposure estimated at exact geocoded addresses. Also, the London study only had information on noise in three categories, and thus less exposure variation than the Nordic studies. As effects of noise on sleep are suspected to be on the pathway between noise and AF, investigating the effect of nighttime noise on AF risk is highly relevant. However, due to a correlation of 0.998 between Lden and Lnight, we were not able to separate the effect of daytime and nighttime exposure in the present study.

Particulate air pollution is a recognized risk factor for AF,^26^ and since road traffic noise and traffic-related air pollution correlate due to mutual emission sources, controlling for traffic related air pollution is crucial. In the present study, estimates were robust to PM_2.5_ adjustment, providing support for road traffic noise as an independent risk factor for AF. Similarly, we observed that although adjustment for NO_2_ reduced the HRs in the analysis without cut-off (from 1.02 to 1.01), NO_2_ adjustment had no effect on the HR in the analysis with a cut-off of 53 dB. Interestingly, we also observed an indication of interaction between particulate air pollution (PM_2.5_) with road traffic noise, with a HR of 1.03 among those exposed to high levels of PM_2.5_ compared to a HR of 1.00 among those with low exposure. Studies have suggested that some biological mechanisms may be similar for air pollution and noise, e.g. inflammation, endothelial dysfunction, and oxidative stress.^27^ However, our finding of interaction should be interpreted with caution, as the cut-point used splits the cohorts into two–those with high (mainly the Danish cohorts) and low (the Swedish and Finnish cohorts) air pollution, and the Danish cohorts show stronger road traffic noise and AF association compared to the other cohorts. Further experimental studies exploring the effects of concomitant exposure to traffic noise and particulate air pollution are needed to elucidate how these exposures interact.

For road traffic noise, we found somewhat stronger associations in individuals with high BMI with some indications of effect modification for other markers of unhealthy lifestyle. Such factors are associated with poor cardiac health and share mechanisms potentially involved in the etiology of AF, such as inflammation and oxidative stress.^27^ Therefore, our study findings suggest that people with unhealthy lifestyles may be more vulnerable to noise, potentially due to already compromised immune and endocrine systems.

We observed no association between railway noise and incident AF. In a study covering all of Denmark, a weak association was observed with railway noise at the most exposed façade (HR: 1.016; 95% CI: 1.006–1.026, per 10 dB), as well as a stronger association with railway noise at the least exposed façade (HR: 1.038; 95% CI: 1.023–1.054, per 10 dB); a noise estimate which was postulated to be a better indicator of nighttime exposure.^3^ If noise at night (bedroom façade) is more harmful than noise during the day, our L_den_ estimates (most exposed façade) were potentially biased towards the null, especially for railway noise. Another likely explanation for our null finding is the limited number of highly exposed persons and, therefore, limited power. Overall, more studies are needed to explore the association between railway noise and AF, especially studies including an estimation of nighttime exposure, most preferable at the bedroom/least exposed facade.

We observed that exposure to aircraft noise appeared to be associated with higher risk of AF, with a tendency towards an exposure-response relationship. Our findings are supported by three prior studies on aircraft noise and AF: a Danish nationwide study observed a slightly higher HR among highly exposed,^3^ a study from Greece showed nighttime aircraft noise to be associated with higher odds of cardiac arrhythmia,^14^ and a cross-sectional study from Germany demonstrated that annoyance from aircraft noise was associated with prevalent AF.^15^ Although further studies on aircraft noise and AF are required, these studies point towards aircraft noise as a contributor to the etiology of AF. Aircraft noise is characterized by a high degree of discontinuity, with many single noise events, whereas road traffic noise is characterized by a low degree of discontinuity. Previous studies have aimed to capture this aspect of noise by estimating a joined road, railway and aircraft noise intermittency.^28^ These studies found that high noise intermittency was associated with a higher risk of cardiovascular mortality.^28^ Our results suggesting that both road traffic and aircraft noise are associated with higher risk of AF calls for more research into the role of noise events in the development of AF.

We found that combined exposure from more than one transportation noise source, thus higher total noise exposure, may be particularly hazardous. Furthermore, multiple noise sources could indicate a lower probability of a quiet façade. Studies have shown that simultaneous exposure to various noise sources impact sleep, annoyance, and cardiometabolic outcomes.^29^^,^^30^ This coincides well with the multiple environmental stressors hypothesis, which purports that several stressors may act cumulatively or synergistically.^31^ Our findings suggest that exposure to multiple noise sources may play an important role for AF and perhaps cardiovascular health in general, but additional studies are required to support these results.

The pooling of 11 Nordic cohorts provided significant strengths to the present study, including a large study with many cases, with harmonized data across the cohorts, with high-quality outcome data from high-quality registries, and with information on various possible lifestyle and sociodemographic confounders, as well as near complete address history for cohort participants through national registries. Moreover, each cohort modeled address level long-term air pollution exposure using validated high-resolution dispersion models. Also, the rather heterogeneous study populations, which included both rural and urban areas from several countries, provided greater generalizability of our results than from an individual cohort.

Some limitations deserve mention. First, although the Nordic prediction method utilizing detailed input data on traffic and screening from topography and buildings was used when estimating road and railway traffic noise for all cohorts, noise was estimated independently for each cohort and did not follow a completely standardized protocol. We did, however, collect detailed information of the noise estimation approach from all cohorts and found some differences between noise assessment across cohorts, including simplifications and differences in underlying GIS data and source inclusion radii. These observed differences in input data and model settings may have resulted in differences in calculated noise levels across cohorts in the low end of the exposure scale (<50 dB), whereas at the higher end of the noise scale, only small differences in calculated noise levels across the cohorts are expected. Second, 37%, of the AF cases originated from the DCH cohort, therefore had a large influence on the results. However, the leave-one-out analyses produced HRs from 1.01 (without DCH) to 1.02 and from 1.03 (without DCH) to 1.04 in analyses using the 53 dB cut-off, indicating that the association was not driven by a single cohort. Third, ascertainment of AF was done through registers, with potential risk of misclassification. For example, it is well-known that people of low socioeconomic position are less likely to use health services and thus less likely to be diagnosed with AF, compared to people of higher socioeconomic position. Although we found no major differences in HRs across people of different educational level, we observed slightly lower risk estimates in people of low educational level, which could potentially be due to underdiagnosis of AF in this group. Furthermore, only three of our 11 cohorts had access to both in- and outpatient records whereas the remaining cohorts relied on inpatient diagnoses only. This is a limitation as higher sensitivity (and comparable specificity) of the AF diagnosis has been found when combining in- and outpatient records compared to inpatient data only.^32^ Our finding of higher risk estimates among the cohorts with access to both in- and outpatient records suggests that underdiagnosis of AF among the cohorts with only inpatient data may have driven the risk estimates towards the null. Fourth, residual confounding is often a concern in epidemiological studies. However, since we observed that increasing levels of adjustment had a limited impact on HRs, this is unlikely to be of large concern in the present study. Fifth, we excluded 8.4% (15,031 persons) of our study base due to missing data one or more covariates, primarily smoking, education and/or physical activity. In analyses including the 11,253 persons excluded due to missing information on one or more of these factors, HRs were unchanged. This suggests that selection bias is not a major concern in the present study. Lastly, we acknowledge that the cause-specific HRs estimated in the current study limits our ability to draw conclusions about the absolute risk of AF in relation to noise.

In this large, pooled Nordic study, residential exposure to road traffic and aircraft noise seemed associated with higher risk of AF, whereas no association with railway noise was observed. Exposure to multiple transportation noise sources seemed particularly harmful. We observed that women and individuals with high BMI seemed more vulnerable to the harmful effect of road traffic noise on AF development. Transportation noise is an increasingly important public health challenge, and our findings lend further support to its role as a risk factor for cardiac health.

## Contributors

MS obtained funding for the study. JDT, MS, NR, AP, TL, MÖ and GP contributed to the study concept and design. MÖ, SSJ, JB, JHC, KE, DS, KM, ES, LaraS, and TY-T participated in modelling of exposure to noise and/or air pollution. KO, MKS, HG, KL, DR, PM, AR, MA, AÅ, GE, and PJ participated in collection of cohort data. NN, AP, AHP, Y-HL, LeoS, EMA, LB, AO, EH, CE, AWT, GE, and PT obtained, cleaned, and/or harmonized cohort data. JDT did the statistical analyses and drafted the paper. JDT, NR, MÖ, AP, AÅ, AO, AR, AWT, AHP, CE, DS, DR, EH, EMA, GMA, GE, HG, JS, JHC, JB, KL, KO, KM, KE, LaraS, LB, LeoS, MA, MKS, ORN, PJ, PT, PLSL, SSJ, SG, TY-T, TCH, TL, Y-HL, ZJA, GP, and MS contributed to a critical revision of the manuscript. JDT has directly accessed and verified the underlying data reported in the manuscript.

## Data sharing statement

The data that support the findings of this study are available at a server at Statistics Denmark. As it includes data from 11 pooled cohorts, data access is only possible for project collaborators approved by all cohort partners and furthermore requires the completion of a Data Processor Agreement as well as an application form to get access to data at Statistics Denmark.

## Ethics approval and consent to participate

Data collection in all included cohorts were approved by relevant national ethics review boards and informed consent was obtained for all participants.

## Declaration of interests

All other authors declare no competing interests.
