# Corrigendum to “Residential exposure to transportation noise and risk of incident atrial fibrillation: a pooled study of 11 prospective Nordic cohorts” [The Lancet Regional Health – Europe, Volume 46, November 2024, 101091]

**DOI:** 10.1016/j.lanepe.2024.101129

**Published:** 2024-11-12

**Authors:** Jesse D. Thacher, Nina Roswall, Mikael Ögren, Andrei Pyko, Agneta Åkesson, Anna Oudin, Annika Rosengren, Aslak H. Poulsen, Charlotta Eriksson, David Segersson, Debora Rizzuto, Emilie Helte, Eva M. Andersson, Gunn Marit Aasvang, Gunnar Engström, Hrafnhildur Gudjonsdottir, Jenny Selander, Jesper H. Christensen, Jørgen Brandt, Karin Leander, Kim Overvad, Kristoffer Mattisson, Kristina Eneroth, Lara Stucki, Lars Barregard, Leo Stockfelt, Maria Albin, Mette K. Simonsen, Ole Raaschou-Nielsen, Pekka Jousilahti, Pekka Tiittanen, Petter L.S. Ljungman, Steen S. Jensen, Susanna Gustafsson, Tarja Yli-Tuomi, Thomas Cole-Hunter, Timo Lanki, Youn-Hee Lim, Zorana J. Andersen, Göran Pershagen, Mette Sørensen

**Affiliations:** a Division of Occupational and Environmental Medicine, Lund University, Lund, Sweden; b Danish Cancer Institute, Strandboulevarden 49, 2100, Copenhagen Ø, Denmark; c Department of Occupational and Environmental Medicine, Sahlgrenska University Hospital, Gothenburg, Sweden; d Occupational and Environmental Medicine, School of Public Health and Community Medicine, Institute of Medicine, University of Gothenburg, Gothenburg, Sweden; e Institute of Environmental Medicine, Karolinska Institutet, Stockholm, Sweden; f Center for Occupational and Environmental Medicine, Region Stockholm, Stockholm, Sweden; g Division of Sustainable Health, Umeå University, Sweden; h Molecular and Clinical Medicine, Sahlgrenska Academy, University of Gothenburg, Gothenburg, Sweden; i Region Västra Götaland, Sahlgrenska University Hospital, Gothenburg, Sweden; j Swedish Meteorological and Hydrological Institute, Norrköping, Sweden; k Aging Research Centre, Department of Neurobiology, Care Sciences and Society, Karolinska Institutet and Stockholm University, Stockholm, Sweden; l Stockholm Gerontology Research Centre, Stockholm, Sweden; m Department of Air Quality and Noise, Norwegian Institute of Public Health, Oslo, Norway; n Department of Clinical Science, Lund University, Malmö, Sweden; o Centre for Epidemiology and Community Medicine, Stockholm, Sweden; p Department of Global Public Health, Karolinska Institutet, Stockholm, Sweden; q Department of Environmental Science, Aarhus University, Roskilde, Denmark; r Department of Public Health, Aarhus University, Aarhus, Denmark; s Environment and Health Administration, Stockholm, Sweden; t Department of Neurology and Parker Institute, Bispebjerg and Frederiksberg Hospital, Denmark; u Department of Public Health and Welfare, Finnish Institute for Health and Welfare, Helsinki, Finland; v Department of Health Security, Finnish Institute for Health and Welfare, Kuopio, Finland; w Department of Cardiology, Danderyd Hospital, Stockholm, Sweden; x Environmental Department of the City of Malmö, Malmö, Sweden; y Section of Environmental Health, Department of Public Health, University of Copenhagen, Copenhagen, Denmark; z School of Medicine, University of Eastern Finland, Kuopio, Finland; aa Department of Environmental and Biological Sciences, University of Eastern Finland, Kuopio, Finland; ab Department of Natural Science and Environment, Roskilde University, Denmark

The authors have noticed an error in Table 2, in which the standard deviations for road traffic noise as well as railway noise were incorrectly presented per 10 dB instead of as raw values. Table 2 has been corrected to show the correct values for standard deviation.

**Table 2:** Baseline exposure to transportation noise (L_den_) and air pollution across the included cohorts.

	DCH	DNC	SDPP	Sixty	SNAC–K	SALT	MDCS	PPS	GOT–MONICA	SMC	FINRISK	Total
**Road traffic noise, 5–y, dB**	56.9 (7.3)	53.9 (8.0)	46.0 (6.4)	51.3 (8.4)	62.2 (6.7)	51.9 (8.5)	54.9 (7.5)	58.2 (7.9)	55.9 (8.0)	54.6 (7.6)	54.4 (7.8)	55.0 (8.0)
**Railway noise, % exposed**^a^	25.9	19.1	14.4	31.6	51.9	34.5	28.1	15.2	19.6	–	34.2	22.7
**Railway noise, 5–y, dB**^b^	52.9 (7.4)	53.1 (7.3)	51.9 (7.4)	50.2 (7.3)	49.7 (5.5)	50.3 (7.5)	49.2 (8.1)	47.0 (5.9)	46.3 (5.4)	–	49.4 (7.2)	51.3 (7.6)
**Aircraft noise, 5–y, %**												
≤40 dB	98.6	98.8	74.7	83.6	15.2	83.3	–	–	–	–	95.6	95.6
40.1–50 dB	0.7	0.4	9.6	13.4	65.3	13.5	–	–	–	–	0.6	2.6
>50 dB	0.7	0.8	15.7	3.0	19.5	3.2	–	–	–	–	3.8	1.8
**PM**_**2.5**_**, 5–y, μg/m**^**3**^	19.8 (1.9)	20.9 (3.4)	7.6 (0.6)	8.1 (0.9)	8.5 (0.8)	7.7 (0.9)	11.0 (0.8)	–	9.3 (1.4)	13.7 (0.8)	7.6 (0.8)	15.4 (5.5)
Missing, %	0.04	9.0	0	0	0	0	0.02	100^c^	0	0.01	0.06	4.7
**NO**_**2**_**, 5–y, μg/m**^**3**^	29.3 (8.1)	13.3 (8.1)	8.8 (2.8)	14.1 (6.7)	21.1 (4.8)	14.2 (6.5)	24.4 (6.4)	31.4 (6.2)	29.2 (7.1)	8.5 (5.9)	16.3 (4.5)	21.1 (10.8)
Missing, %	0.04	9.0	0	0	0	0	0.02	2.5	0	0.01	0.06	1.6

Values given as mean and standard deviation (SD) unless otherwise stated.

a Exposed in the 5-year period preceding baseline.

b Among exposed to railway noise.

c PPS only had information on PM_2.5_ from baseline until follow–up.

However, the mean values are accurate, so the authors consider that these changes do not affect the interpretation of the findings or the conclusions.

The authors would like to apologise for any inconvenience caused.

