# Supplementary material for: Residential exposure to transportation noise and risk of incident atrial fibrillation: a pooled study of 11 prospective Nordic cohorts
Source: Lancet Reg Health Eur. 2024 Oct 1;46:101091. doi: 10.1016/j.lanepe.2024.101091 (PMC11472630; doi:10.1016/j.lanepe.2024.101091)

Supplementary Material

Residential exposure to transportation noise and risk of incident atrial fibrillation: a pooled study of 11 Nordic cohorts

Jesse Thacher et al.

Supplementary Table 1 **Page 2**
Detailed information on the participating cohorts

Supplementary Table 2
Detailed information on estimation of road traffic noise for all participating cohorts **Page 5**

Supplementary Table 3 **Page 7**
Detailed information on estimation of railway traffic noise for all participating cohorts.

Supplementary Table 4 **Page 8**
Detailed information on estimation of air pollution for all participating cohorts.

Supplementary Table 5 **Page 10**
Characteristics of the study population across participating cohorts at the NordSOUND baseline.

Supplementary Table 6 **Page 12**
Associations between 5-year mean traffic noise and risk of AF in sensitivity analyses with further adjustments to adjustment model 3.

Supplementary Table 7 **Page 13**
Association between exposure to 1-year time-weighted traffic noise and risk of atrial fibrillation.

Supplementary Table 8 **Page 14**
Association between 5-year time-weighted traffic noise (per 10 dB) and atrial fibrillation incidence by cohort.

Supplementary Table 9 **Page 15**
Association between 5-year mean exposure to road traffic noise (per 10 dB) and risk of atrial fibrillation following one-by-one exclusion of each cohort.

Supplementary Table 10 **Page 16**
Association between 5–year mean exposure to road traffic noise and risk of atrial fibrillation in complete case analyses compared to analyses including cohort participant excluded from main study population due to lack of co-variates

Supplementary Table 11 **Page 17**
Associations between combined exposure (5-year) to road traffic (≥45 dB), railways (≥40 dB), and/or aircraft (≥40 dB) and risk of atrial fibrillation.

Supplementary Table 12 **Page 18**
Interactions between 5-year road traffic noise exposure (per 10 dB) and demographic and lifestyle-factors in relation to atrial fibrillation incidence.

Supplementary Table 13 **Page 19**
Interactions between 5-year road traffic noise exposure (per 10 dB) and demographic and lifestyle-factors in relation to atrial fibrillation incidence – *using a 53 dB cut-off.*

Supplementary Figure 1 **Page 20**
Directed Acyclic Graph displaying the relationship between transportation noise, atrial fibrillation, and other variables.

Supplementary Table 1. Detailed information on the participating cohorts.

Cohort	Detailed cohort information	Key references	Funding and acknowledgments
**DCH**	The inclusion criteria for the Danish Diet Cancer and Health (DCH) cohort were age between 50 and 64 y, residing in the greater Copenhagen or Aarhus area and without a cancer diagnosis. From 1993 to 1997, 160,725 Danes were invited to participate of whom 57,053 participants accepted the invitation and were enrolled in the study. All participants completed detailed questionnaires at enrolment and trained staff members measured height, weight, and waist circumference. End of follow-up for atrial fibrillation was 31 Dec 2016.	Tjonneland A, Olsen A, Boll K, et al. Study design, exposure variables, and socioeconomic determinants of participation in Diet, Cancer and Health: a population-based prospective cohort study of 57,053 men and women in Denmark. Scand J Publ Health 2007;35:432-41 DOI: 10.1080/14034940601047986	The Danish Cancer Society
**DNC**	The Danish Nurse Cohort (DNC) was initiated by sending questionnaires to the members of the Danish Nurse Organization in 1993 and 1999. Among 33,704 eligible female nurses aged 44-93 years who either worked or were retired in 1993 or 1999, 28,731 participants (85·2%) were included in the DNC. Upon enrolment, participants answered a comprehensive questionnaire on body mass index (BMI), lifestyle factors (smoking, alcohol consumption, physical activity, and dietary habits), self-reported diseases and reproductive health, and working conditions. End of follow-up for atrial fibrillation was 31 Dec 2014.	Hundrup, Yrsa A, Jørgensen T, Obel EB. Cohort profile: the Danish nurse cohort. Int J Epidemiol 2012; 41:1241-47. DOI: 10.1093/ije/dyr042	The Danish Council for Independent Research (DFF-4183-00353).
**SDPP**[a]	The Stockholm Diabetes Preventive Programme (SDPP) is a prospective cohort aimed at investigating the aetiology of type 2 diabetes and cardiometabolic risk factors. The cohort comprises 34 486 men and women aged 35-56 years who lived in five municipalities in Stockholm County 1992-1998. A sub-sample of 7,949 individuals were invited to a clinical examination at baseline and were followed up 2002-2006 and 2014-2017. During the clinical examinations, blood pressure was measured along with anthropometric measurements, blood samples were collected, oral glucose tolerance tests were administered, and participants answered extensive questionnaires concerning lifestyle factors, health status, socioeconomic characteristics, and psychosocial conditions.	Gudjonsdottir H, Tynelius P, Fors S, et al. Cohort Profile: The Stockholm Diabetes Prevention Programme (SDPP). International Journal of Epidemiology. 2022. DOI: 10.1093/ije/dyac147	Swedish Environmental Protection Agency, the Swedish Council for Health, Working Life and Social Research and the Swedish Heart-Lung Foundation. The SDPP cohort was additionally funded by the Stockholm County Council, the Swedish Research Council, the Swedish Diabetes Association and Novo Nordisk Scandinavia.
**SIXTY**[a]	The SIXTY cohort is based on a random sample of every third man and woman living in Stockholm County, who were born in 1937 and 1938. A total of 4,232 subjects were recruited 1997-1999 to investigate risk factors for cardiovascular disease. People were 59-61 years at enrolment. Measurements of anthropometric indices and blood pressure were made at recruitment and fasting blood samples were collected. In addition, a comprehensive questionnaire was completed, including information on socioeconomic, medical and lifestyle factors. End of follow-up for atrial fibrillation was 31 Dec 2016.	Wändell PE, Wajngot A, de Faire U, et al. Increased prevalence of diabetes among immigrants from non-European countries in 60-year- old men and women in Sweden. Diabetes Metab 2007;33:30–6. DOI: 10.1016/j.diabet.2006.07.001	Swedish Environmental Protection Agency, the Swedish Council for Health, Working Life and Social Research and the Swedish Heart- Lung Foundation. The SIXTY cohort was additionally funded by the Stockholm County Council and the Swedish Research Council.
**SALT**[a]	The Screening Across the Lifespan Twin Study (SALT) included a total of about 45 000 twins born 1958 and earlier from the Swedish Twin Registry who were interviewed 1998-2002. Those 7,043 who resided in Stockholm County at recruitment are included in the present project. People were 42-97 years at enrolment. The interview collected data on zygosity, diseases, use of medication, occupation, education and lifestyle habits. In a subgroup of around 2,500 subjects, a clinical examination was made, including blood sampling and anthropometrics as well as blood pressure measurements. End of follow-up for atrial fibrillation was 31 Dec 2017.	Lichtenstein P, Sullivan PF, Cnattingius S, et al. The Swedish Twin Registry in the third millennium: an update. Twin Res Hum Genet 2006;9:875–82. DOI: 10.1017/thg.2012.104	Swedish Environmental Protection Agency, the Swedish Council for Health, Working Life and Social Research and the Swedish Heart- Lung Foundation. The SALT cohort was additionally supported by NIH grant 575 AG-08724. The Swedish Twin Registry is managed by Karolinska Institutet and receives funding through the Swedish Research Council under the grant no 2017-00641.
**SNAC-K**[a]	The Swedish National Study of Aging and Care in Kungsholmen (SNAC-K) was established 2001-2004 and included 3,363 residents aged 60-104 years in Kungsholmen, Stockholm. The aim was to investigate the ageing process and identify possible	Lagergren M, Fratiglioni L, Hallberg IR, et al. A longitudinal study integrating population, care and social services data. The	Swedish Environmental Protection Agency, the Swedish Council for Health, Working Life, the Swedish Research Council and Social Research

	preventive strategies to improve health and care in elderly adults. Information was collected through social interviews and clinical examinations, including assessment of physical and cognitive functioning.<br>Follow-up investigations are performed at intervals of three to six years depending on age. End of follow-up for atrial fibrillation was 31 Dec 2016.	Swedish National study on Aging and Care (SNAC). Aging Clin Exp Res 2004;16:158–68.<br>DOI: 10.1007/BF03324546	and the Swedish Heart- Lung Foundation. SNAC-K was additionally funded by the Ministry of Health and Social Affairs, Sweden, Stockholm County Council and the participating Municipalities and University Departments.
**PPS**	The Primary Prevention Study cohort (PPS) consists of a random third of all men in the city of Gothenburg born 1915–1925, recruited in 1970–1973 (n=7,494, participation rate 75%) to study predictors of cardiovascular disease. People were 46-55 years at enrolment. Participants were examined by health care professionals (e.g. height, weight, systolic and diastolic blood pressures and cholesterol levels) and filled out questionnaires on background data (e.g. occupation, smoking habits, physical activity, antihypertensive medication, psychological stress, prevalent diabetes mellitus and family history of coronary events). End of follow-up for atrial fibrillation was 31 Dec 2011.	Wilhelmsen L, Tibblin G, Werkö L. A primary preventive study in Gothenburg, Sweden. Preventive Med. 1972;1:153-60.<br>Wilhelmsen L, Berglund G, Elmfeldt D et al. The multifactor primary prevention trial in Göteborg, Sweden. Eur Heart J. 1986;7:279-88.<br>DOI: 10.1093/oxfordjournals.eurheartj.a062065	The Bank of Sweden Tercentenary Fund and the Swedish Medical Research Council. The Swedish Research Council 2019-00193.
**GOT-MONICA**	The GOT-MONICA cohort is part of the “Multinational Monitoring of Trends and Determinants in Cardiovascular Diseases” (MONICA) project. A random selection of residents in Gothenburg aged 25–64 years at the time of inclusion were recruited in the years 1985, 1990, and 1995 (participation rates: 63%, 69%, and 72%). The participants filled out questionnaires on e.g. smoking habits, physical activity, hypertensive medication, psychological stress, marital status, and were examined by health care professionals (e.g. blood pressures, height and weight). End of follow-up for atrial fibrillation was 31 Dec 2011.	Wilhelmsen L, Johansson S, Rosengren S et al. Risk factors for cardiovascular disease during the period 1985–1995 in Goteborg, Sweden. The GOT-MONICA Project. J. Intern Med, 1997;242:199- 211.<br>DOI: 10.1046/j.1365-2796.1997.00163.x	The Swedish Medical Research Council, the Swedish Heart and Lung Foundation, the Ingabritt and Arne Lundberg Research Fund, the Göteborg Medical Society and the Sahlgrenska University Hospital Funds. The Swedish Research Council 2019-00193.
**MDCS**	The Malmö Diet and Cancer (MDCS) study is a population based prospective cohort study. People were enrolled into the cohort between 1991 and 1996, and eligible participants were men born between 1923 and 1945 and women born between 1923 and 1950, living in the city of Malmö. Swedish reading and writing skills were required. People were 44-73 years at enrolment. The data collection was done both using questionnaires and interviews, including data on dietary habits, socio-economics, medical history and lifestyle factors. The total number of study subjects were 28,098. End of follow-up for atrial fibrillation was 31 Dec 2016.	Berglund G, Elmstahl S, Janzon L et al. The Malmö Diet and Cancer study. Design and feasibility. J Intern Med 1993;233:45–51.<br>DOI: 10.1111/j.1365-2796.1993.tb00647.x<br>Manjer J, Carlsson S, Elmstahl S et al. The Malmö Diet and Cancer Study: representativity, cancer incidence and mortality in participants and non-participants. Eur J Cancer Prev, 2001;10:489–499.<br>DOI: 10.1097/00008469-200112000-00003	Swedish Research Council (VR) Infrastructure grant, Heart-Lung Foundation.
**SMC**	The Swedish Mammography Cohort, part of SIMPLER (Swedish Infrastructure for Medical Population-based Life-course and Environmental Research; www.simpler4 health.se), a population-based prospective cohort study was established 1987-90 when all 90,303 women living in Uppsala and Västmanland counties, born 1914-48, were invited to a mammography screening and to respond to a questionnaire (74% response rate). In 1997, the 56,030 participants who were still living in the study area were invited to respond to a 2nd extended questionnaire, (response rate 70 %). In the present project, we included the women from Uppsala County taking part in the 1997 investigation (n= 20,407) women for residential exposure to air pollution, road traffic noise and urban greenness.	Harris H, Håkansson N, Olofsson C, Stackelberg O, Julin B, Åkesson A, Wolk A. The Swedish mammography cohort and the cohort of Swedish men: study design and characteristics of two population based longitudinal cohorts. OA Epidemiology, 2013 Oct 01;1(2):16.<br>DOI: 10.13172/2053-079X-1-2-943	We acknowledge SIMPLER for provisioning of facilities and experimental support. SIMPLER receives funding through the Swedish Research Council under the grant no 2017-00644.
**FINRISK**	The national FINRISK study is a set of large population surveys that have been conducted to monitor non-communicable disease risk factors, health behavior and their changes in the population. Every five years during 1972-2012 a stratified random sample has been selected from the 25-74 (64 in earlier surveys) year old inhabitants in different regions of Finland. For the NordSOUND study data from four surveys (1997, 2002, 2007, 2012) and	Borodulin K, Tolonen H, Jousilahti P et al. Cohort Profile: The National FINRISK Study. Int J Epid, 2018;47:696-696i.<br>DOI: 10.1093/ije/dyx239	Funding was mostly provided by the Ministry of Social Affairs and Health / Finnish Institute for Health and Welfare

	two study areas (the cities of Helsinki/Vantaa in Southern Finland and Turku region in Southwestern Finland). The age group 25 to 74 years is covered otherwise except that for 1997 and 2002 the age group is 25 to 64 years in the Turku region. The surveys included a self-administered questionnaire and a clinical examination with measurements of height, weight and blood pressure and blood sampling. End of follow-up for atrial fibrillation was 31 December 2015.		The questionnaire data used for the research was obtained from THL Biobank (study numbers BB2019_13 and THLBB2021_8). We thank all study participants for their generous participation at THL Biobank and the National FINRISK Study. Statistics Finland provided data on causes of death and socioeconomic factors. Hospital discharge data was extracted from Care Register for Health Care administered by Finnish Institute for Health and Welfare.

[a] This cohort is part of The Swedish Cardiovascular Effects of Air Pollution and Noise in Stockholm (CEANS) cohort, which consists of four sub-cohorts of persons residing in Stockholm County, Sweden. Harmonization of covariates has been conducted across the cohorts.

Supplementary Table 2. Detailed information on estimation of road traffic noise for all participating cohorts.

Cohort	Road traffic noise estimation	Key references
**DCH**	Calculations were conducted for the years 1995, 2000, 2005, 2010, and 2015 using the Nordic prediction method implemented in SoundPLAN (version 8·0). Various input variables were used in the model, most importantly geocode and height (floor) for each address; information on travel speed, light/heavy vehicle distributions, road type, annual average daily traffic for all Danish road links (Jensen et al 2019) and 3D information on all Danish buildings. Screening effects from buildings, terrain, and noise barriers were included. All road traffic sources within 1500 m from the receivers were included. The parameter setting were set to allow 2 reflections.	Thacher JD, Poulsen AH, Raaschou-Nielsen O, et al. High-resolution assessment of road traffic noise exposure in Denmark. Environ Res 2019; 182:109051<br>Jensen SS, Plejdrup MS, Hillig K. GIS-based National Road and Traffic Database 1960- 2020. Aarhus University, Danish Centre for Environment and Energy 2019; Report 151<br><br>B Plovsing, J Kragh (2006) Nord2000. Validation of the propagation model. Delta Acoustics and Vibration Report vol. AV 1117/06
**DNC**	Same method as for DCH.	
**CEANS (SDPP, SIXTY, SALT, SNAC-K)**	To assess long-term individual transportation noise exposure a noise database for Stockholm County was developed representing the period from 1990 and onwards, with detailed estimation every fifth year. The database includes 3D terrain data as well as information on ground surface, road net, daily traffic flows, speed limits and percentage of heavy vehicles. To calculate noise levels for road traffic a modification of the Nordic prediction method was used, where possible reflection and shielding were taken into account by a Ground Space Index based on building density. The methodology has been developed from the one described by Ögren and Barregard (2016), which was validated against the full Nordic prediction method modelled with SoundPlan and showed coherent estimates.	Ringheim, M. The new Nordic prediction method for railway noise. Journal of sound and vibration 193.1 (1996): 277-282<br><br>Ögren M, Barregard L. Road traffic noise exposure in Gothenburg 1975-2010. PLoS One. 2016;11:e015532.
**PPS, GOT- MONICA**	Yearly average road traffic flows, speed and percentage of heavy vehicles were obtained from the environmental office of the municipality of Gothenburg and the traffic office of the municipality of Mölndal. The traffic flow estimations were based on measurements for all major and medium links but used a standard default flow for very small streets. Terrain data and building footprints were obtained from Lantmäteriet and road links from the Swedish National Traffic Administration. Noise barriers of at least 2 m height and 100 m length were also included, and earth berms were included in the terrain model. To save calculation time and reduce demands on detailed input data a simplified methodology was used for multiple reflections in dense urban areas. The methodology has been developed from the one described by Ögren and Barregard (2016), which was validated against the full Nordic prediction method modelled with SoundPlan and showed coherent estimates.	Ögren M, Barregard L. Road traffic noise exposure in Gothenburg 1975-2010. PLoS One. 2016;11:e015532.
**MDCS**	Estimated for the years 1990, 2000 and 2010, using the Nordic Prediction Method implemented in SoundPLAN (version 8·0, SoundPLAN Nord ApS). Input variables included geocode, information on annual average daily traffic for all road links in Malmö municipality, distribution of light/heavy traffic, signposted travel speed and road type and polygons for all buildings in Malmö. All road traffic sources within 1000m from the receivers were included. Traffic data were retrieved from a regional emission database (Rittner et al. 2020). The screening effects from buildings were included and ground softness considered. Terrain was not included, as Malmö is relatively flat. The parameter setting in the models were set to allow 2 reflections and receivers placed at 2m height. Linear interpolation was not used for intermediate years but instead used the three models from 1990, 2000 and 2010 and addressed the exposure based on the year closest in time or the year of major changes in infrastructure. For road traffic the model from 1990 were used for 1985-1999, the model 2000 used 2000-2005 and the model from 2010 between 2006 and 2016.	Ringheim, M. The new Nordic prediction method for railway noise. Journal of sound and vibration 193.1 (1996): 277-282<br><br>Rittner R, Gustafsson S, Spanne M, Malmqvist E. Particle concentrations, dispersion modelling and evaluation in southern Sweden, SN Applied Sciences 2020;2:1013.
**SMC**	Exposure to road traffic noise was assessed using the Nordic Prediction Method (Bendtsen 1999). Input data included ground surface (assuming flat terrain), road net and traffic flows (simulated and calibrated against historical measurements) on both state owned and municipal roads, diurnal distributions, percentage heavy vehicles, speed, and buildings. The exposure was calculated as free-field levels at the façade of the buildings at 2 m height. Within urban areas (primarily within the city of Uppsala), the calculations were performed with second-order reflections, whereas in more rural areas, first-order of reflections were used. The search radii were set to 1,000 m within Uppsala and to	Bendtsen, H., 1999. The nordic prediction method for road traffic noise. Sci. Total Environ. 235, 331–338. https://doi.org/10.1016/S0048-9697(99)00216-8.

	1,500 m in more rural areas. The estimated sound parameter, Lden, was calculated for each address every fifth year from 1990 to 2015. To calculate yearly averages, we applied linear interpolation.	
**FINRISK**	Façade noise levels from road-traffic were calculated by consulting companies in accordance with the EU Environmental Noise Directive 2002/49/EC50 using input data for the year 2011. The Nordic Prediction Method was used for major highways, main streets, and collector streets within Helsinki, Vantaa, and Turku. Input variables included terrain elevation data, traffic flows on the road network, speed limits and percentage of heavy vehicles, locations and heights of noise barriers. In addition, bridges, road profiles as well as acoustic hardness of terrain or water surfaces were specified. All road traffic sources within 2000 m from the receivers were included. Counting height was 4 m. The parameter setting were set to allow 1 reflection, but not from the facade in question. No weather corrections were used. The highest Lden on façade points within 20 m of residential address coordinates was assigned as the noise exposure to that dwelling.	The Assessment of Road and Railway Traffic Noise of Finnish Transport Agency 2012. Finnish Transport Agency, Transport System. Helsinki 2012. 34 pages. ISBN 978-952-255-158-0.<br><br>Road-traffic and Rail-traffic Noise Assessment of the Finnish Transport Agency 2017. Finnish Transport Agency, Engineering and Environment. Helsinki 2017. 25 pages and 1 appendix. ISBN 978-952-317-507-5.<br><br>Sito Oy. 2012. Pääkaupunkiseudun ympäristömeludirektiivin mukainen meluselvitys. https://docplayer.fi/6614750-Paakaupunkiseudun-ymparistomeludirektiivin-mukainen-meluselvitys-yhdistelmaraportti.html<br><br>Pöyry Finland Oy. 2012. Ympäristömeludirektiivin mukainen ympäristömeluselvitys Turussa. https://www.turku.fi/sites/default/files/atoms/files//ymparistomeluselvitys_turussa_loppuraportti_1.6.2012.pdf

Supplementary Table 3. Detailed information on estimation of railway traffic noise for all participating cohorts.

Cohort	Railway noise estimation	Key references
**DCH**	A total noise model with all railways, buildings, and terrain was prepared for both 1997 and 2012. Input variables included geographical coordinates for each address, building polygons and building heights, noise barriers and year of construction, and information on annual average daily train length, train type and weighted speed. Absorption or reflection from terrain was also considered. All rail sources within 1000 m from the receivers were included. The parameter setting in the models were set to allow for two reflections. The calculations were made in accordance with NORD2000 for railway traffic noise and the noise calculation program SoundPLAN version 8.0, was used for the calculations.	B Plovsing, J Kragh (2006) Nord2000. Validation of the propagation model. Delta Acoustics and Vibration Report vol. AV 1117/06
**DNC**	Same method as for DCH.	
**CEANS (SDPP, SIXTY, SALT, SNAC-K)**	To assess long-term individual transportation noise exposure a noise database for Stockholm County was developed representing the period from 1995 and onwards, with detailed estimation every fifth year. The database includes 3D terrain data as well as information on ground surface, rail network, daily traffic, speed limits and vehicle types. To calculate noise levels a modification of the Nordic prediction method was used, where possible reflection and shielding were considered by a Ground Space Index based on building density. The methodology has been developed from the one described by Ögren and Barregard (2016), which was validated against the full Nordic prediction method modelled with SoundPlan and showed coherent estimates.	Ringheim, M. The new Nordic prediction method for railway noise. Journal of sound and vibration 193.1 (1996): 277-282<br><br>Ögren M, Barregard L. Road traffic noise exposure in Gothenburg1975-2010. PLoS One. 2016;11:e015532.
**MDCS**	Estimated for the years 1990, 2000 and 2010, using the Nordic Prediction Method implemented in SoundPLAN (version 8.0, SoundPLAN Nord ApS). Input variables included geocode, information on annual average daily traffic for all railway lines in Malmö municipality, distribution of different rail vehicles and train types, maximum speed and polygons for all buildings in Malmö. All rail sources within 1000 m from the receivers were included. The screening effects from buildings were included and ground softness considered, but the terrain was assumed to be flat in order to reduce calculation time. The parameter setting in the models were set to allow 2 reflections and receivers placed at 2m height. The model from 1990 was assigned to address exposure between 1985-1999, the model from 2000 between 2000-2009 and the model 2010 between 2010-2016. The reason for using these breaks were that the Öresund bridge were completed in 2000 and the City-tunnel in 2010.	Ringheim, M. The new Nordic prediction method for railway noise. Journal of sound and vibration 193.1 (1996): 277-282
**FINRISK**	Façade noise levels from major railways (> 30.000 train passages a year) were calculated by consulting companies in accordance with the EU Environmental Noise Directive 2002/49/EC50 using input data for the year 2011 for trains, and year 2016 for trams and underground. In the 2012 assessment, the Nordic Prediction Method was used for train traffic in Helsinki, Vantaa and Turku, while the Common Noise Assessment Methods in Europe (CNOSSOS-EU) was used in the 2017 assessment for trams and underground in Helsinki. Input variables included traffic data (number of passages, types and lengths of trains, speed), terrain elevation data, buildings, and locations and heights of noise barriers. In addition, acoustic hardness of terrain or water surfaces were specified. All rail traffic sources within 2000 m from the receivers were included. Counting height was 4 m. The parameter settings were set to allow 1 reflection, but not from the facade in question. The highest L_{den} on façade points within 20 m of residential address coordinates was assigned as the noise exposure to that dwelling.	The Assessment of Road and Railway Traffic Noise of Finnish Transport Agency 2012. Finnish Transport Agency, Transport System. Helsinki 2012. 34 pages. ISBN 978-952-255-158-0.<br><br>Road-traffic and Rail-traffic Noise Assessment of the Finnish Transport Agency 2017. Finnish Transport Agency, Engineering and Environment. Helsinki 2017. 25 pages and 1 appendix. ISBN 978-952-317-507-5.

Supplementary Table 4. Detailed information on estimation of air pollution for all participating cohorts.

Cohort	Air pollution estimation	Key references
**DCH**	In the DCH cohort, we used the DEHM-UBM-AirGIS modelling system to calculate $PM_{2.5}$ and NO_2 at all Danish addresses for the years 2000, 2010 and 2015, which was then extrapolated to yearly means for each address, based on changes in yearly urban background levels. This multi-scale dispersion modelling system calculates air pollutants at each address as the sum of: a) $PM_{2·5}$/NO_2 from the nearest street, calculated based on traffic, car fleet emission factors, streets and building geometry, and meteorology; b) urban background, calculated based on city dimensions, emission density, and heights of buildings; and c) regional background, calculated based on all emissions in the northern hemisphere.	Khan J, Kakosimos K, Raaschou-Nielsen O, et al. Development and performance evaluation of new AirGIS – A GIS based air pollution and human exposure modelling system. Atmos Environ 2019;198:102-121.<br>Ketzel M, Berkowicz R, Hvidberg M, Jensen SS and Raaschou-Nielsen O. Evaluation of AirGIS - a GIS-based air pollution and human exposure modelling system. Int J Environ Pollution 2011;47:226–238.<br>www.au.dk/AirGIS
**DNC**	Same method as for DCH.	
**CEANS (SDPP, SIXTY, SALT, SNAC-K)**	In the Stockholm County cohorts, a high-resolution Gaussian dispersion model was used to estimate individual residential levels of $PM_{2·5}$ and NO_x/NO_2 using local emission inventories every fifth year from 1990 and onwards. The emission inventory contains detailed information on local emissions from road and ferry traffic, industrial areas and households. Meteorological input to the modelling includes measurements of wind velocity and direction, solar radiation and temperature. Further, a street canyon contribution is added for addresses in the most polluted street segments of the inner city of Stockholm with multi-storey houses on both sides. Annual averaged long-range contributions were added to the locally modelled concentrations based on continuous measurements at regional background monitoring stations.	Segersson D, Eneroth K, Gidhagen L, et al. Health impact of PM10, PM2.5 and black carbon exposure due to different source sectors in Stockholm, Gothenburg and Umea, Sweden. Int J Environ Res Public Health 2017;14:E742.<br>Ljungman PLS, Andersson N, Stockfelt L, et al. Long-Term Exposure to Particulate Air Pollution, Black Carbon, and Their Source Components in Relation to Ischemic Heart Disease and Stroke. Environ Health Perspect 2019;127:107012
**PPS**	The exposure assessment was performed similarly for Gothenburg and Stockholm as part of the Swedish Clean air and Climate Research program (SCAC). For Gothenburg, high-resolution dispersion modeling of source-specific particulate matter <2·5 µm ($PM_{2·5}$), and nitrogen oxides (NO_X – converted to NO_2) was performed over an area of 93 × 112 km for the years 1990, 2000 and 2011. Emission inventories were compiled using local and regional bottom-up inventories provided by the municipality, and supplemented to be consistent for the whole time-period. Intervening years were interpolated so that each participant could be assigned annual residential air pollutant exposures. For NO_2 additional data were available from dispersion modelling for the period before 1990, as described in Stockfelt et al. 2015.	Segersson D, Eneroth K, Gidhagen L et al. Health impact of PM10, $PM_{2.5}$ and black carbon exposure due to different source sectors in Stockholm, Gothenburg and Umea, Sweden. Int J Environ Res Public Health, 2017;14:E742.<br>Stockfelt L, Andersson EM, Molnár P, et al. Long-term effects of total and source-specific particulate air pollution on incident cardiovascular disease in Gothenburg, Sweden. Environ Res. 2017;158:61-71.
**GOT- MONICA**	Same method as for PPS.	
**MDCS**	Air pollutants ($PM_{2·5}$, and nitrogen oxides (NO_X) – converted to NO_2) were modelled using EnviMan (Opsis AB, Sweden) by the Environmental Department, City of Malmö, using a Gaussian dispersion model (AERMOD) combined with an emission database for the county of Scania in Sweden. The 18 × 18 km modelling area covered the city of Malmö and the closest surroundings. Separate emission databases were compiled for 1992, 2000 and 2011 using existing local and regional bottom-up inventories provided by the municipality, and then supplemented to be consistent for the whole area and time-period. Yearly mean concentrations were stored as grids with a spatial resolution of 50 m × 50 m. The years in between the modelled years were interpolated linearly with adjustment for year-to-year variations in the local meteorology using a ventilation factor estimated from calculations over the whole time-period, and exposure for the years 1990 and 1991 extrapolated. Exposure data was combined with geocoded addresses to assign each participant annual residential exposures.	Xu Y, Andersson EM, Krage Carlsen H, Molnár P, Gustafsson S, Johannesson S, Oudin A, Engström G, Christensson A, Stockfelt L: Associations between long-term exposure to low-level air pollution and risk of chronic kidney disease—findings from the Malmö Diet and Cancer cohort. Environment International 2022, 160:107085.<br><br>Rittner R, Gustafsson S, Spanne M, Malmqvist E: Particle concentrations, dispersion modelling and evaluation in southern Sweden. SN Applied Sciences 2020, 2:1-15.<br><br>Azzouz M, Xu Y, Barregard L, Zöller B, Molnar P, Oudin A, Spanne M, Engström G, Stockfelt L: Long-term ambient air pollution and venous thromboembolism in a population-based Swedish cohort. Environmental pollution 2023, 331:121841.<br><br>Xu Y, Andersson EM, Krage Carlsen H, Molnár P, Gustafsson S, Johannesson S, Oudin A, Engström G, Christensson A, Stockfelt L: Associations between long-term exposure to low-level air pollution and risk of chronic kidney

		disease—findings from the Malmö Diet and Cancer cohort. Environment International 2022, 160:107085.
**SMC**	Exposure to air pollution was assessed by SLB Analys (https://www.slb.nu/slbanalys/) using a dispersion modelling (Segersson et al. 2017). Sources considered were emissions from road traffic (including street canyon effect where applicable), boilers and energy plants, individual heating with solid fuel (wood) and oil, shipping, and long-range transport. Yearly average concentrations of $PM_{2.5}$, PM_{10} and NO_2 for all sources combined were calculated for all addresses of our study participants.	Segersson D, Eneroth K, Gidhagen L et al. Health impact of PM10, $PM_{2.5}$ and black carbon exposure due to different source sectors in Stockholm, Gothenburg and Umea, Sweden. Int J Environ Res Public Health, 2017;14:E742.
**FINRISK**	Estimates of $PM_{2.5}$ and NO_2 concentrations were based on dispersion modelling using models developed in the Finnish Meteorological Institute. The calculations included emissions from energy production, industry, ship traffic and road traffic. The average measured background was added to the concentrations. Emissions used in the calculations represented the situation in Turku Region in 2007 and 2014 in the Helsinki Capital Region. In the dispersion models, the distances between the receptor points varied from 25 m near the roads to 500 m in rural areas. In the present study, the modelled annual average concentration at the nearest outdoor receptor point was used as a proxy of home outdoor concentration.	Salmi, J., Lappi, S., Rasila, T., Lovén, K. ja Hannuniemi, H. 2009. Turun seudun päästöjen leviämismalliselvitys. Finnish Meteorological Institute. Helsinki. http://expo.fmi.fi/aqes/public/Turun_seudun_leviamismallilaskelmat_2010.pdf<br><br>Hannuniemi, H., Salmi, J., Rasila, T., Wemberg, A., Komppula, B., Lovén, K. ja Pietarila, H. 2016. Pääkaupunkiseudun päästöjen leviämismalliselvitys. Finnish Meteorological Institute. Helsinki. http://expo.fmi.fi/aqes/public/PKS_Ilmanlaaturaportti_01.06.2016.pdf<br><br>Aarnio MA, Kukkonen J, Kangas L, Kauhaniemi M, Kousa A, Hendriks C, Yli-Tuomi T, Lanki T, Hoek G, Brunekreef B: A Model Evaluation Strategy Applied to Modelling of PM in the Helsinki Metropolitan Area. In: Air Pollution Modeling and its Application XXV 35: 2018: Springer; 2018: 103-109.<br><br>Srimath ST, Sokhi R, Karppinen A, Singh V, Kukkonen J: Evaluation of an urban modelling system against three measurement campaigns in London and Birmingham. Atmospheric Pollution Research 2017, 8(1):38-55.<br><br>Kukkonen J, Harkonen J, Walden J, Karppinen A, Lusa K: Validation of the dispersion model CAR-FMI against measurements near a major road. International journal of environment and pollution 2001, 16(1-6):137-147.

Supplementary Table 5. Characteristics of the study population across participating cohorts at the NordSOUND baseline.

	DCH	DNC	SDPP	Sixty	SNAC-K	SALT	MDCS	PPS	GOT-MONICA	SMC	FINRISK	TOTAL
**Enrolment area**	Copenhagen Aarhus	Denmark	Stockholm county	Stockholm county	Stockholm city	Stockholm county	Malmö	Gothenburg	Gothen-burg	Västman-land, Uppsala county	Finland	--
**Total participants, N[a]**	54,452	26,450	7,522	3,889	2,482	6,323	27,316	5,276	2,342	17,057	8,006	161,115
**Enrolment period [b]**	1993–1997	1993, 1999	1992–1998	1997–1999	2001–2004	1998–2002	1991–1996	1970–1973	1985, 1990, or 1995	1987–1990	1997–2012	—
**End of follow–up**	2016	2014	2011	2016	2016	2017	2016	2011	2011	2017	2015	—
**Follow-up time, years**	20·0 (5·9–21·8)	17·4 (5·9–21·0)	15·0 (12·1–18·7)	18·3 (6·2–19·0)	12·5 (1·2–15·1)	16·0 (4·0–18·5)	19·2 (5·3–22·7)	25·0 (11·8–36·8)	13·0 (6·8–18·0)	20·2 (4·5–20·2)	8·8 (2·0–18·7)	19·6 (4·9–22·3)
**Atrial fibrillation cases, N**	6,922	2,201	224	564	585	852	3,280	1136	129	2,629	417	18,939
**Sex, %**												
Men	47·3	0	39·4	47·7	38·8	45·1	39·0	100	47·5	0	46·5	34·3
Women	52·7	100	60·6	52·3	61·2	54·9	61·0	0	52·5	100	53·5	65·7
**Age at inclusion**	56·3 (50·9–64·4)	50·9 (45·0–71·7)	48·0 (38·0–53·9)	60·4 (60·3–60·8)	72·4 (60·4–90·5)	56·5 (44·5–79·6)	58·0 (47·3–71·6)	55·0 (49·8–60·0)	—[e]	59·9 (49·8–78·6)	51·1 (28·2–70·8)	55·9 (45·0–72·0)
**Educational level, %**												
Low	27·9	0	31·5	39·8	23·3	27·0	68·1	68·4	20·7	37·7	25·0	32·6
Medium	48·8	100	38·6	32·4	39·7	36·6	17·6	20·3	50·2	34·2	53·8	48·2
High	23·3	0	29·9	27·9	37·0	36·5	14·3	11·3	29·1	28·1	21·2	19·2
**Marital status, %**												
Married/cohabiting	76·8	70·4	83·5	74·2	48·6	67·6	65·4	85·8	69·0	76·3	70·0	72·9
**Area–level income, %**												
1st quartile	33·1	32·8	3·5	4·5	3·0	7·1	23·3	25·8	22·0	25·2	19·3	25·9
2nd quartile	22·4	26·6	5·7	8·7	0	10·5	21·0	22·3	15·1	22·5	25·9	21·0
3rd quartile	16·9	24·8	21·0	24·3	0·2	18·6	26·6	24·6	21·4	27·1	26·8	21·9
4th quartile	27·6	15·8	69·9	62·5	96·8	63·9	29·1	27·3	41·4	22·2	28·0	31·2
**Smoking Status, %**												
Current	36·4	35·3	26·3	21·1	15·1	20·3	28·3	40·0	28·0	22·9	26·7	31·1
Former	28·1	30·6	36·4	38·8	40·2	36·2	33·7	33·3	23·8	23·7	29·1	30·3
Never	35·5	34·1	37·3	40·1	44·7	43·5	38·0	26·7	48·2	53·4	44·2	38·6
**Physical activity, %**												
Low	51·7	6·9	65·8	69·4	74·6	54·8	50·4	25·3	17·6	20·3	17·5	39·3
Medium	19·5	66·4	26·5	23·2	18·6	36·1	21·0	59·2	62·8	24·3	32·3	31·6
High	28·8	26·7	7·7	7·4	6·8	9·1	28·6	15·5	19·6	55·4	50·1	29·1
**BMI (kg/m^2)**	25·5 (20·4–33·3)	23·1 (19·2–30·2)	25·1 (20·4–33·3)	26·2 (21·0–34·3)	25·3 (19·9–32·8)	24·2 (19·6–30·6)	25·3 (20·2–33·0)	25·1 (20·7–30·5)	24·5 (19·7–32·3)	24·3 (19·7–32·0)	25·9 (20·3–35·0)	24·9 (19·9–32·7)
**Smoking intensity, g/day[c,d]**	15·1 (5·0–32·1)	15·0 (3·0–25·0)	15·0 (2·0–25·0)	13·0 (2·0–25·0)	10·0 (0–30·0)	10·0 (2·0–25·0)	14·0 (1·0–30·0)	—	15·0 (4·0–25·0)	10·0 (2·0–20·0)	12·0 (1·6–25·0)	14·4 (3–30)
Missing, %	0·7	1·9	0	0	1·9	0	0	100	0	12·6	9·6	6·2

**Alcohol intake, %** [c]												
Daily	19·8	12·8	4·0	5·7	11·4	9·0	16·6	—	1·0	1·2	15·5	13·9
Weekly	59·1	60·0	65·1	40·0	47·9	63·0	34·3	—	35·4	34·3	46·7	51·0
Seldom	18·3	11·5	27·5	44·8	33·9	25·0	32·6	—	56·3	46·4	29·9	25·5
Never	2·8	15·8	3·4	9·5	6·8	3·0	16·5	—	7·3	18·1	7·8	9·6
Missing	0·06	2·5	0·3	0·001	0	0·003	1·6	100	0·7	2·9	1·0	4·4
**Population density, %** [c]												
Low	7·2	45·9	37·0	8·6	— [e]	6·1	0	0	— [e]	21·9	0	14·5
Medium	56·5	22·0	36·5	18·5	— [e]	16·9	0	0·5	0·6	20·9	0	27·8
High	36·3	30·9	26·5	72·9	99·9	77·0	100	99·3	98·3	55·9	100	57·4
Missing	0	1·2	0	0	0	0	0	0·2	1·2	1·4	0	0·3

Median and 5–95 percentiles, unless otherwise stated.

[a] Only participants with full information on exposure variables, outcome, and covariates included in Model 2.

[b] Original enrolment period for each cohort.

[c] Not available for all.

[d] Among current smokers.

[e] Too few individuals to report.

Supplementary Table 6. Associations between 5-year mean traffic noise and risk of AF in sensitivity analyses with further adjustments to adjustment model 3.

	**Persons with information on smoking intensity and alcohol**		**Persons with information on 5-year mean exposure to NO_2**		**Persons with information on 5-year mean exposure to $PM_{2·5}$**		**Adjustment for BMI**	
	**Model 3[a]**	**Model 3[a] plus smoking intensity and alcohol adjustment [b]**	**Model 3[a]**	**Model 3[a] plus adjustment for NO2**	**Model 3[a]**	**Model 3[a] plus adjustment for PM2·5**	**Model 3[a]**	**Model 3 plus adjustment for BMI**
	**HR (95% CI)**	**HR (95% CI)**	**HR (95% CI)**	**HR (95% CI)**	**HR (95% CI)**	**HR (95% CI)**	**HR (95% CI)**	**HR (95% CI)**
**Road traffic noise**								
N, cases	14,766	14,766	18,070	18,070	17,999	17,999	18,939	18,939
5-year mean per 10 dB	1·03 (1·01–1·05)	1·03 (1·01–1·05)	1·02 (1·00–1·04)	1·01 (0·99–1·04)	1·02 (1·00–1·04)	1·02 (1·00–1·04)	1·02 (1·00–1·04)	1·02 (1·00–1·04)
5-year exposure, per 10 dB with 53 dB cut-off	1·04 (1·01–1·07)	1·04 (1·01–1·07)	1·03 (1·00–1·06)	1·03 (0·99–1·06)	1·03 (1·00–1·06)	1·03 (1·00–1·06)	1·03 (1·01–1·06)	1·03 (1·00–1·06)
**Railway noise**								
N, cases	14,766	14,766	18,070	18,070	17,999	17,999	18,939	18,939
5-year mean per 10 dB	0·97 (0·93–1·01)	0·97 (0·93–1·01)	0·98 (0·94–1·02)	0·98 (0·94–1·02)	0·98 (0·94–1·02)	0·98 (0·94–1·02)	0·97 (0·93–1·01)	0·97 (0·93–1·01)
**Aircraft noise**								
≤ 40 dB	Reference	Reference	Reference	Reference	Reference	Reference	Reference	Reference
40·1–50 dB	1·04 (0·93–1·17)	1·03 (0·92–1·16)	1·04 (0·93–1·17)	1·04 (0·93–1·17)	1·04 (0·93–1·17)	1·04 (0·93–1·17)	1·04 (0·93–1·16)	1·03 (0·92–1·15)
≥ 50 dB	1·13 (0·99–1·30)	1·13 (0·99–1·29)	1·12 (0·99–1·27)	1·12 (0·99–1·27)	1·12 (0·99–1·27)	1·12 (0·99–1·27)	1·12 (0·98–1·27)	1·13 (0·99–1·28)

HR: Hazard Ratio; 95% CI: 95% Confidence Interval

[a] Model 3: adjusted for age, cohort, sex, calendar year, educational level, marital status, area-income, other noise sources (road/rail, continuous), railway noise (yes/no), aircraft noise (≤40 dB; 40–50 dB, >50 dB; for the 3 cohorts without aircraft noise information all cohort members were assigned to the ≤40 dB group), smoking status, and physical activity

[b] Model 3 plus adjustment for smoking intensity (g/day, continuous) and alcohol consumption (daily, weekly, seldom, never).

Supplementary Table 7. Association between exposure to 1-year time-weighted traffic noise and risk of atrial fibrillation.

	N cases	Model 1[a] HR (95% CI)	Model 2[b] HR (95% CI)	Model 3[c] HR (95% CI)	Model 4[d] HR (95% CI)	Model 5[e] HR (95% CI)
**Road traffic noise, L_{den}**						
1-year exposure, per 10 dB	18,939	1·03 (1·01–1·04)	1·02 (1·00–1·04)	1·02 (1·00–1·04)	1·02 (1·00–1·04)	1·01 (0·99–1·03)
1-year exposure, per 10 dB with 53 dB cut-off	6,784 <53 dB	1·04 (1·02–1·07)	1·03 (1·01–1·06)	1·03 (1·00–1·06)	1·03 (1·00–1·06)	1·02 (0·99–1·06)
**Railway noise, L_{den}**						
1-year exposure, per 10 dB	18,939	0·98 (0·94–1·02)	0·97 (0·93–1·01)	0·97 (0·93–1·01)	0·98 (0·94–1·02)	0·98 (0·94–1·02)
**Aircraft noise, L_{den}, 1-year[f]**						
≤ 40 dB	10,977	Reference	Reference	Reference	Reference	Reference
40·1–50 dB	489	1·07 (0·96–1·20)	1·07 (0·95–1·20)	1·07 (0·96–1·20)	1·08 (0·96–1·20)	1·07 (0·96–1·20)
>50 dB	299	1·08 (0·95–1·23)	1·09 (0·96–1·24)	1·09 (0·96–1·24)	1·10 (0·97–1·24)	1·10 (0·97–1·24)

HR: Hazard Ratio; 95% CI: 95% Confidence Interval.

[a] Model 1: adjusted for age, cohort (strata), sex and calendar year.

[b] Model 2: Model 1 plus adjustment for educational level, marital status, area-income, and other noise source (road, railway, and aircraft noise), for the four cohorts without aircraft noise information, all cohort members were assigned to the ≤40-dB group).

[c] Model 3: Model 2 plus adjustment for smoking status, and physical activity.

[d] Model 4: Model 3 plus adjustment for time-weighted $PM_{2.5}$ exposure (1-year). $PM_{2.5}$ exposure history available for 17,999 cases

[e] Model 5: Model 2 plus adjustment for time-weighted NO_2 exposure (1- year).

[f] Only among cohorts with aircraft noise-exposure, which includes 11,765 cases.

Supplementary Table 8. Association between 5-year time-weighted traffic noise (per 10 dB) and atrial fibrillation incidence by cohort.

	DCH	DNC	SDPP	Sixty	SNAC-K	SALT	MDCS	PPS	GOT-MONICA	SMC	FINRISK
**Road traffic noise, L_{den} Model 3b[a]**											
N cases	6,922	2,201	224	564	585	852	3280	1136	129	2629	417
HR (95% CI), per 10 dB	1·03 (0·99–1·06)	1·05 (0·99–1·10)	1·03 (0·85–1·25)	0·93 (0·84–1·03)	0·93 (0·82–1·06)	1·08 (0·99–1·18)	1·01 (0·96–1·06)	0·92 (0·84–1·01)	1·26 (0·98–1·61)	1·01 (0·96–1·06)	1·03 (0·91–1·18)
N cases <53 dB	1966	901	166	313	44	390	1270	246	34	983	202
HR (95% CI), per 10 dB with 53 dB cut-off	1·03 (0·98–1·08)	1·09 (1·00–1·19)	0·70 (0·40–1·20)	0·85 (0·69–1·05)	0·96 (0·81–1·13)	1·26 (1·09–1·47)	1·01 (0·93–1·09)	0·91 (0·80–1·03)	1·31 (0·91–1·90)	1·04 (0·95–1·13)	0·94 (0·74–1·19)
**Railway noise, L_{den} Model 3b[a]**											
HR (95% CI), per 10 dB	0·96 (0·90–1·03)	1·03 (0·91–1·17)	0·81 (0·49–1·31)	0·94 (0·77–1·15)	1·23 (0·99–1·52)	0·95 (0·81–1·10)	0·95 (0·89–1·02)	1·19 (0·91–1·55)	0·68 (0·29–1·60)	–	0·84 (0·65–1·08)
**Aircraft noise, HR (95% CI), L_{den} Model 3b[a]**											
≤ 40 dB	Reference	Reference	Reference	Reference	Reference	Reference	–	–	–	–	Reference
40·1–50 dB	1·09 (0·80–1·49)	0·62 (0·23–1·65)	0·89 (0·57–1·39)	1·29 (0·98–1·69)	1·01 (0·80–1·27)	1·11 (0·89–1·37)	–	–	–	–	0·87 (0·12–6·31)
≥ 50 dB	1·15 (0·88–1·51)	1·03 (0·64–1·66)	1·30 (0·73–2·33)	1·36 (0·88–2·09)	1·21 (0·94–1·56)	0·92 (0·63–1·32)	–	–	–	–	0·87 (0·53–1·43)

HR: Hazard Ratio; 95% CI: 95% Confidence Interval

[a] Adjusted for age, sex, calendar year, educational level, marital status, area-income, smoking status, physical activity, and other noise sources (road/rail, continuous), railway noise (yes/no); aircraft noise (≤40 dB; 40–50 dB, >50 dB; for the four cohorts without aircraft noise information all cohort members were assigned to the ≤40 dB group)

Supplementary Table 9. Association between 5-year mean exposure to road and railway noise (per 10 dB) and risk of atrial fibrillation following one-by-one exclusion of each cohort.

		Road traffic noise		Railway noise	
**Cohort excluded**	N cases	Model 3[a] HR (95% CI)	Model 3[a] HR (95% CI) with 53 dB cut-off	Model 3[a] HR (95% CI)	Model 3[a] HR (95% CI) with 53 dB cut-off
**DCH**	12,017	1·01 (0·99–1·04)	1·03 (0·99–1·06)	0·98 (0·93–1·03)	0·95 (0·87–1·04)
**MDCS**	15,659	1·02 (1·00–1·04)	1·03 (1·00–1·06)	0·98 (0·93–1·02)	0·96 (0·88–1·05)
**DNC**	16,738	1·02 (0·99–1·04)	1·02 (1·00–1·05)	0·97 (0·93–1·01)	0·95 (0·88–1·02)
**SDPP**	18,715	1·02 (1·00–1·04)	1·03 (1·00–1·06)	0·97 (0·94–1·01)	0·96 (0·89–1·03)
**SALT**	18,087	1·02 (1·00–1·04)	1·03 (1·00–1·06)	0·97 (0·93–1·01)	0·96 (0·89–1·03)
**SIXTY**	18,375	1·02 (1·00–1·04)	1·04 (1·01–1·06)	0·97 (0·93–1·01)	0·95 (0·88–1·02)
**SNAC-K**	18,354	1·02 (1·00–1·04)	1·04 (1·01–1·07)	0·97 (0·93–1·00)	0·94 (0·87–1·01)
**GOT-MONICA**	18,810	1·02 (1·00–1·04)	1·03 (1·00–1·06)	0·97 (0·93–1·01)	0·95 (0·89–1·02)
**PPS**	17,803	1·02 (1·00–1·04)	1·04 (1·01–1·07)	0·97 (0·93–1·01)	0·95 (0·88–1·02)
**FINRISK**	18,522	1·02 (1·00–1·04)	1·03 (1·01–1·06)	0·98 (0·94–1·02)	0·95 (0·89–1·02)
**SMC**	16,310	1·02 (1·00–1·04)	1·03 (1·00–1·06)	–	–

[a] Adjusted for age, sex, calendar year, educational level, marital status, area-income, smoking status, physical activity, and other noise sources (road/rail, continuous), railway noise (yes/no); aircraft noise (≤40 dB; 40–50 dB, >50 dB; for the four cohorts without aircraft noise information all cohort members were assigned to the ≤40 dB group.

Supplementary Table 10. Association between 5–year mean exposure to road traffic noise and risk of atrial fibrillation in complete case analyses compared to analyses including cohort participant excluded from main study population due to lack of co-variates

**Road traffic noise, L_{den} 5–year, per 10 dB**	**N cases / N population**	**Model 1** Adjusted age, cohort, sex, year **HR (95% CI)**	**Model 2** Model 1 + adjustment for co-variates **HR (95% CI)**	**Model 3** Model 2 + adjustment for $PM_{2.5}$ **HR (95% CI)**
Complete case analysis (=all population)	18,939 / 161,115	1·03 (1·01–1·05)	1·02 (1·00–1·04)[a]	1·02 (1·00–1·04)
Complete case analysis (=all population)	18,939 / 161,115	1·03 (1·01–1·05)	1·02 (1·00–1·04)[b]	1·02 (1·00–1·04)
All population + inclusion of participants lacking information on education, smoking and/or physical activity	19,981 / 172,368	1·03 (1·01–1·05)	1·02 (1·00–1·04)[b]	1·02 (1·00–1·04)

HR: Hazard Ratio; 95% CI: 95% Confidence Interval.

[a] Model 1 + adjustment for educational level, marital status, area–income, other noise sources (railway and aircraft noise), smoking status and physical activity.

[b] Model 1 + adjustment for marital status, area–income, and other noise sources (railway and aircraft noise).

Supplementary Table 11. Associations between combined exposure (5-year) to road traffic (≥45 dB), railways (≥40 dB), and/or aircraft (≥40 dB) and risk of atrial fibrillation.

	N cases	HR (95% CI)[b]
**No elevated traffic noise exposure [a]**	1,429	1·00 (ref)
**1 noise source**	12,584	1·04 (0·98–1·10)
**2 noise sources**	4,665	1·06 (1·00–1·13)
**3 noise sources**	261	1·19 (1·02–1·40)

HR: Hazard Ratio; 95% CI: 95% Confidence Interval

[a] No road traffic noise ≥45 dB, no railway noise >40 dB, no aircraft noise >40 dB

[b] Adjusted for age, cohort, sex, calendar year, educational level, marital status, area-income, smoking, and physical activity

Supplementary Table 12. Interactions between 5-year road traffic noise exposure (per 10 dB) and demographic and lifestyle-factors in relation to atrial fibrillation incidence.

	N cases	Model 3 [a,b] HR (95% CI)	P for Interaction [c]
**Sex**			0·06
Men	8,385	1·00 (0·97–1·03)	
Women	10,554	1·04 (1·01–1·06)	
**Age at diagnosis**			NA
<60 years	1,025	1·04 (0·96–1·13)	
60–80 years	12,964	1·01 (0·99–1·04)	
≥80 years	4,950	1·03 (0·99–1·07)	
**Educational level**			0·87
Low	7,513	1·01 (0·98–1·04)	
Medium	8,228	1·02 (1·01–1·05)	
High	3,198	1·02 (0·98–1·07)	
**BMI**			<0·01
< 25 kg/m^2	7,763	0·98 (0·95–1·00)	
25–30 kg/m^2	7,903	1·03 (1·00–1·06)	
≥ 30 kg/m^2	3,273	1·09 (1·06–1·12)	
**Physical activity**			0·20
Low	8,031	1·03 (1·00–1·06)	
Medium	5,340	1·03 (1·00–1·07)	
High	5,568	0·99 (0·96–1·03)	
**Smoking status**			0·11
Never	7,247	0·99 (0·96–1·02)	
Former	6,034	1·04 (1·01–1·07)	
Current	5,658	1·03 (0·99–1·06)	
**$PM_{2·5}$ (5-year)**			0·16
Low (<11·8 µg/m^3)	8,982	1·00 (0·97–1·03)	
High (≥11·8 µg/m^3)	9,017	1·03 (1·00–1·06)	
**Railway exposure**			0·29
>53 dB	1,074	1·07 (0·98–1·16)	
≤53 dB	17,865	1·02 (1·00–1·04)	
**Population density**			0·43
Low	2,255	1·03 (1·00–1·07)	
Medium	5,062	1·00 (0·96–1·04)	
High	11,548	1·01 (0·99–1·04)	
**Previous ischemic heart disease**			0·22
Yes	2,881	1·04 (1·00–1·09)	
No	16,058	1·01 (0·99–1·03)	
**AF diagnosis based on**			NA
Inpatient data	6,536	1·00 (0·96–1·03)	
In- and outpatient data	12,403	1·03 (1·01–1·05)	

[a] Adjusted for age, cohort, sex, calendar year, educational level, marital status, area-income, smoking status, physical activity, and other noise sources (road and rail (continuous), railway noise (yes/no), aircraft noise (≤40 dB; 40–50 dB, >50 dB, for the 3 cohorts without aircraft noise information all cohort members were assigned to the ≤40 dB group)
[b] All analyses included an interaction term between the potential effect modifiers and the 5-year mean noise exposure
[c] Interaction tested by Wald test

Supplementary Table 13. Interactions between 5-year road traffic noise exposure (per 10 dB) and demographic and lifestyle-factors in relation to atrial fibrillation incidence – *using a 53 dB cut-off.*

	N cases	Model 3[a,b] HR (95% CI)	P for interaction[c]
**Sex**			0·23
Men	8,385	1·02 (0·98–1·06)	
Women	10,554	1·05 (1·02–1·09)	
**Age at diagnosis**			NA
<60 years	1,025	0·92 (0·71–1·18)	
60–80 years	12,964	1·03 (1·00–1·06)	
≥80 years	4,950	1·05 (1·00–1·11)	
**Educational level**			0·92
Low	7,513	1·04 (1·00–1·08)	
Medium	8,228	1·04 (1·00–1·08)	
High	3,198	1·02 (0·96–1·09)	
**BMI**			<0·01
< 25 kg/m^2	7,763	1·00 (0·96–1·03)	
25–30 kg/m^2	7,903	1·01 (1·00–1·08)	
≥ 30 kg/m^2	3,273	1·10 (1·06–1·14)	
**Physical activity**			0·43
Low	8,031	1·05 (1·01–1·09)	
Medium	5,340	1·04 (0·99–1·10)	
High	5,568	1·01 (0·96–1·06)	
**Smoking status**			0·16
Never	7,247	1·00 (0·96–1·05)	
Former	6,034	1·06 (1·01–1·11)	
Current	5,658	1·04 (1·00–1·09)	
**$PM_{2·5}$ (5-year)**			0·22
Low (<11·8 µg/m^3)	8,982	1·01 (0·97–1·06)	
High (≥11·8 µg/m^3)	9,017	1·05 (1·01–1·09)	
**Railway exposure**			0·24
>53 dB	1,074	1·07 (0.98–1.16)	
≤53 dB	17,865	1·02 (1.00–1.04)	
**Population density**			0·78
Low	2,255	1·03 (0·99–1·07)	
Medium	5,062	1·01 (0·95–1·06)	
High	11,548	1·03 (0·99–1·06)	
**Previous IHD**			0·18
Yes	2,881	1·07 (1·00–1·14)	
No	16,058	1·02 (0·99–1·05)	
**AF diagnosis based on**			NA
Inpatient data	6,536	1·01 (0·96–1·06)	
In- and outpatient data	12,403	1·04 (1·01–1·08)	

[a] Adjusted for age, cohort, sex, calendar year, educational level, marital status, area-income, smoking status, physical activity, and other noise sources (road and rail (continuous), railway noise (yes/no), aircraft noise (≤40 dB; 40–50 dB, >50 dB, for the 3 cohorts without aircraft noise information all cohort members were assigned to the ≤40 dB group)
[b] All analyses included an interaction term between the potential effect modifiers and the 5-year mean noise exposure
[c] Interaction tested by Wald test

Supplementary Figure 1. Directed Acyclic Graph displaying the relationship between transportation noise, atrial fibrillation, and other variables.

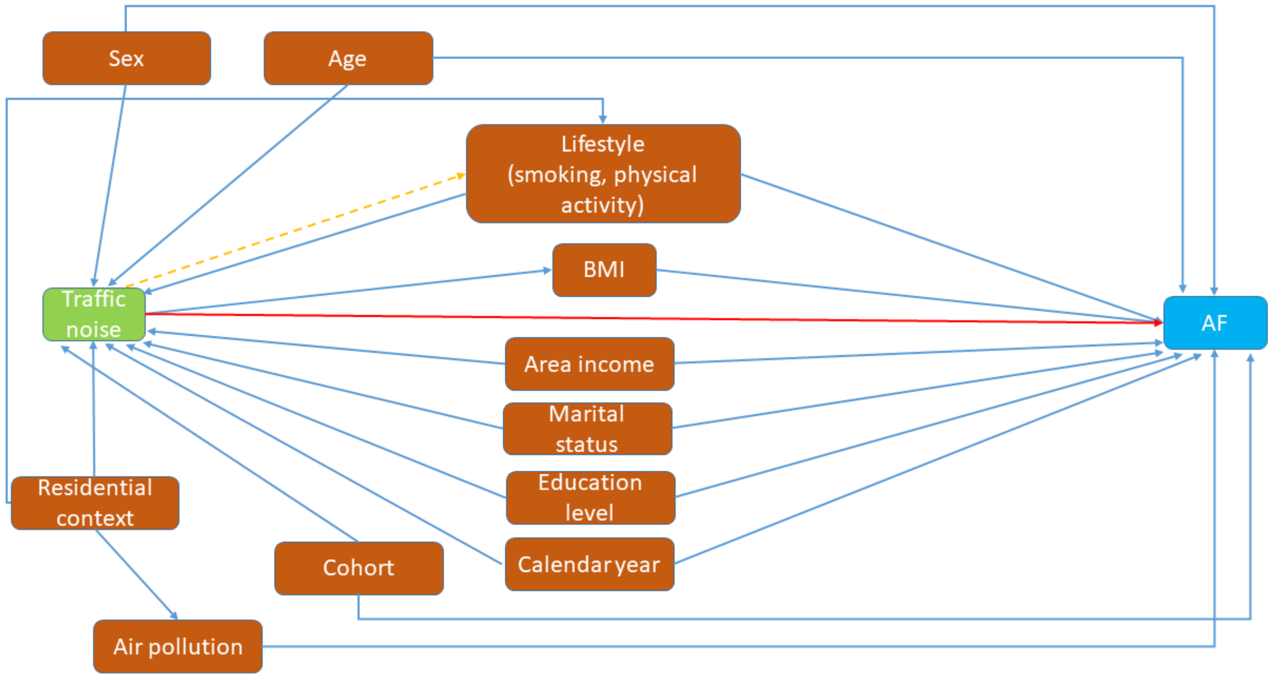

Supplement: Supplementary Tables and Figure [file mmc1.pdf]
